# Elucidation of *Zymomonas mobilis* physiology and stress responses by quantitative proteomics and transcriptomics

**DOI:** 10.3389/fmicb.2014.00246

**Published:** 2014-05-22

**Authors:** Shihui Yang, Chongle Pan, Gregory B. Hurst, Lezlee Dice, Brian H. Davison, Steven D. Brown

**Affiliations:** ^1^Biosciences Division, Oak Ridge National LaboratoryOak Ridge, TN, USA; ^2^BioEnergy Science Center, Oak Ridge National LaboratoryOak Ridge, TN, USA; ^3^National Bioenergy Center, National Renewable Energy LaboratoryGolden, CO, USA; ^4^Computer Science and Mathematics Division, Oak Ridge National LaboratoryOak Ridge, TN, USA; ^5^Chemical Sciences Division, Oak Ridge National LaboratoryOak Ridge, TN, USA

**Keywords:** *Zymomonas mobilis*, microarray, proteomics and metabolomics, acetate, pretreatment inhibitor, stress responses, quantitative proteomics, systems biology

## Abstract

*Zymomonas mobilis* is an excellent ethanologenic bacterium. Biomass pretreatment and saccharification provides access to simple sugars, but also produces inhibitors such as acetate and furfural. Our previous work has identified and confirmed the genetic change of a 1.5-kb deletion in the sodium acetate tolerant *Z. mobilis* mutant (AcR) leading to constitutively elevated expression of a sodium proton antiporter encoding gene *nhaA*, which contributes to the sodium acetate tolerance of AcR mutant. In this study, we further investigated the responses of AcR and wild-type ZM4 to sodium acetate stress in minimum media using both transcriptomics and a metabolic labeling approach for quantitative proteomics the first time. Proteomic measurements at two time points identified about eight hundreds proteins, or about half of the predicted proteome. Extracellular metabolite analysis indicated AcR overcame the acetate stress quicker than ZM4 with a concomitant earlier ethanol production in AcR mutant, although the final ethanol yields and cell densities were similar between two strains. Transcriptomic samples were analyzed for four time points and revealed that the response of *Z. mobilis* to sodium acetate stress is dynamic, complex, and involved about one-fifth of the total predicted genes from all different functional categories. The modest correlations between proteomic and transcriptomic data may suggest the involvement of posttranscriptional control. In addition, the transcriptomic data of forty-four microarrays from four experiments for ZM4 and AcR under different conditions were combined to identify strain-specific, media-responsive, growth phase-dependent, and treatment-responsive gene expression profiles. Together this study indicates that minimal medium has the most dramatic effect on gene expression compared to rich medium followed by growth phase, inhibitor, and strain background. Genes involved in protein biosynthesis, glycolysis and fermentation as well as ATP synthesis and stress response play key roles in *Z. mobilis* metabolism with consistently strong expression levels under different conditions.

## Background

Yeast strains are among the leading current generation industrial biocatalyst microorganisms for fuel production (Hahn-Hagerdal et al., 2006). However, engineered bacteria such as *Zymomonas mobilis*, *E. coli, Bacillus subtilis* are also being developed and deployed to address commercial biofuel catalyst requirements (Dien et al., 2003; Inui et al., 2004; Romero et al., 2007; Alper and Stephanopoulos, 2009). *Z. mobilis* is an ethanologenic bacterium with many desirable industrial characteristics such as high-specific productivity and yield, high ethanol tolerance, and wide pH range (Gunasekaran and Raj, 1999; Panesar et al., 2006; Rogers et al., 2007). Recently, transformation efficiency has been improved by modifying the DNA restriction-modification systems (Kerr et al., 2010), and the inhibitor tolerance genes have been identified to improve the pretreatment inhibitor tolerance using genes from *Z. mobilis* (Yang et al., 2010a,b) or from *Deinococcus radiodurans* (Zhang et al., 2010). The genome sequences for strains ZM4, NCIMB 11163, 10988, 29291 and 29292 have been determined (Seo et al., 2005; Kouvelis et al., 2009, 2011; Pappas et al., 2011; Desiniotis et al., 2012), and the ZM4 genome annotation was improved recently (Yang et al., 2009a). Genome-scale *in silico* metabolic modeling analysis have been reported (Lee et al., 2010; Widiastuti et al., 2011; Rutkis et al., 2013) and recombinant strains have been engineered to express and secret cellulase (Linger et al., 2010) or ferment hexoses and pentose sugars such as xylose and arabinose (Zhang et al., 1995; Deanda et al., 1996).

A core challenge in next-generation biomass-based cellulosic biofuel is the recalcitrance of biomass to breakdown into sugars (Himmel et al., 2007; Alper and Stephanopoulos, 2009). Biomass pretreatment regimes are required to release the sugars, which can create inhibitors such as furfural, acetate, and vanillin (Almeida et al., 2007; Pienkos, 2009). The existence of pretreatment inhibitors increases production costs due to lower production rates and decreased yields. The development and deployment of robust inhibitor-tolerant biocatalysts for efficient fermentation of biomass to biofuel will be a critical component for successful production of biofuels at industrial-scale quantities to meet sustainability and energy security challenges associated with fossil fuels (Almeida et al., 2007). Several recent transcriptomics studies have used microarray approach to characterize mutant strain or *Z. mobilis* stress responses (Yang et al., 2009b, 2013; Hayashi et al., 2011; He et al., 2012a,b; Jeon et al., 2012; Skerker et al., 2013).

Acetic acid is an important inhibitor produced by the de-acetylation of hemicelluloses during biomass pretreatment. Unlike another major inhibitor furfural which is volatile and can be converted into a less toxic product of furfural alcohol (Liu et al., 2005; Heer and Sauer, 2008; Franden et al., 2009; Agrawal and Chen, 2011), acetate is stable during fermentation and poses a constitutive stress on the growth and ethanol production of *Z. mobilis* (Yang et al., 2010a,b). A *Z. mobilis* mutant strain, designated AcR, generated by chemical mutagenesis and selection, is able to produce ethanol efficiently in the presence of 20 g/L sodium acetate (NaAc), while the parent ZM4 is inhibited significantly above 12 g/L (Joachimstahl et al., 1998). Through comparative genome sequencing and next-generation sequencing (NGS)-based genome resequencing, we characterized the AcR mutant and identified a 1.5-kb deletion in strain AcR, which likely truncated the promoter of the *nhaA* gene encoding a sodium proton antiporter. We have carried out genetics study to confirm the association of 1.5-kb deletion in AcR mutant with its sodium acetate tolerance phenotype, we further performed microarray study to identify the differentially expressed genes between wild-type ZM4 and AcR mutant, and identified that *nhaA* gene is consistently upregulated in AcR mutant background compered to wild-type ZM4 under different conditions of NaCl and NaAc stress (Yang et al., 2010b).

Although we confirmed the 1.5-kb deletion in AcR mutant background leading to *nhaA* gene overexpression for enhanced sodium acetate tolerance phenotype (Yang et al., 2010b), we haven't systematically explored the global transcriptional profile difference between AcR mutant and wild-type ZM4 especially in the condition of minimal medium (MM), which is potentially more relevant to industrial fermentation conditions and more stressful to *Z. mobilis* than rich media (RM) we used for previous study (Yang et al., 2010b). In addition, there is no systems biology for this important industrial strain in minimal medium yet. To further explore the acetate-tolerance differences between ZM4 and AcR in MM condition, comprehensive microarray-based transcriptomic profiles and ^14/15^N-labelled quantitative proteomic data were generated for ZM4 and AcR in MM with a large number of *Z. mobilis* proteins detected and quantified, which will be useful for future biocatalyst development. In addition, data collected in this and previous studies were used to hypothesize condition-responsive (strain, media, growth phase, or treatment) genes.

## Results

### Physiological response of *Z. mobilis* to sodium acetate in minimal medium (MM)

The growth of *Z. mobilis* wild-type ZM4 and AcR mutant in MM supplemented with 0, 12, or 16 g/L NaAc as well as 8.65 or 11.4 g/L NaCl with same corresponding Na^+^ molar concentrations as NaAc was assessed using a Bioscreen C instrument (Growth Curves USA, NJ) under anaerobic conditions to determine the effect of NaCl and NaAc on *Z. mobilis* growth and to decide on an appropriate NaAc concentration for subsequent systems biology studies. *Z. mobilis* grew more slowly in MM and attained lower cell densities (Additional File 1) compared to that in RM conditions (Yang et al., 2010b). Consistent with earlier RM results, wild-type ZM4 growth was arrested when NaAc was added to minimum medium at 16 g/L, and differences were observed between ZM4 and AcR with NaAc supplemented at concentrations of 12 g/L (Additional File 1). The concentration of 10 g/L was chosen for NaAc treatment in subsequent systems biology studies.

**Figure 1:**
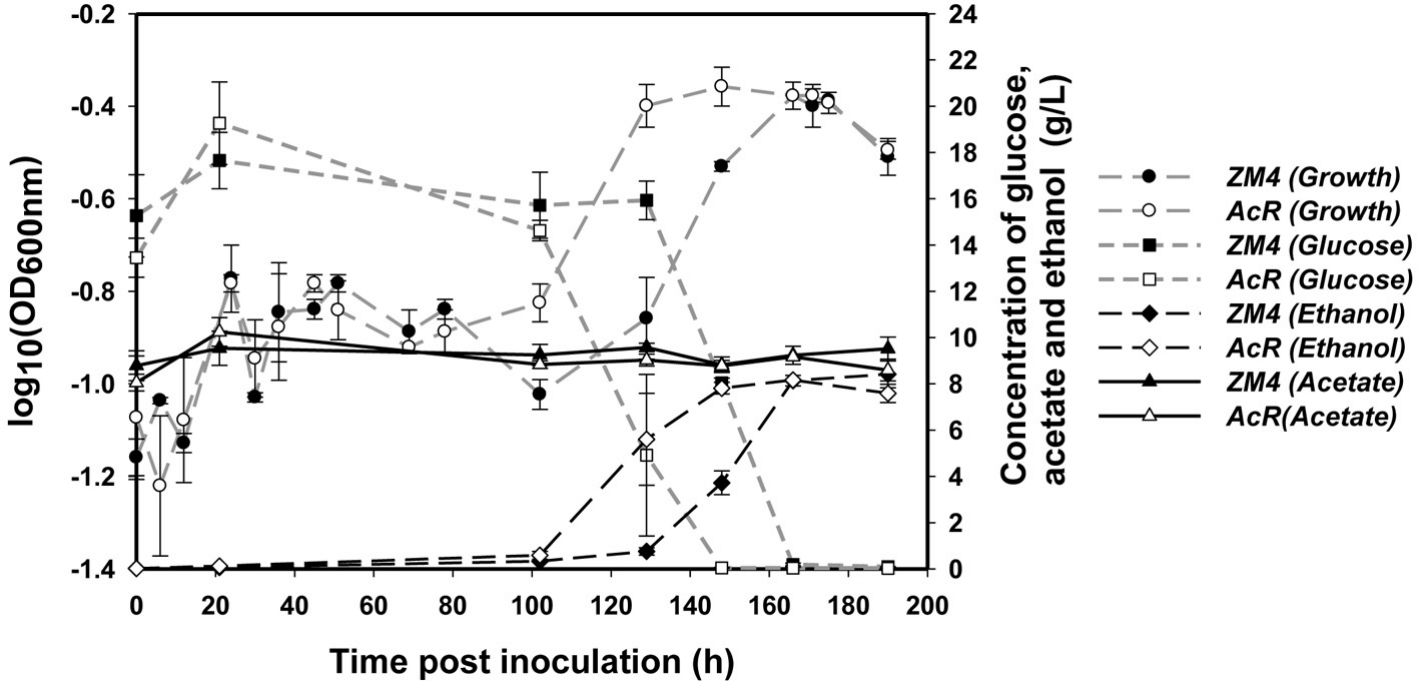
**The growth, glucose consumption, ethanol production and acetate amount of *Z. mobilis* wild-type ZM4 and acetate-tolerant mutant AcR in the presence of ~10 g/L NaAc**.

Similar to previous reports in RM (Joachimstahl et al., 1998; Yang et al., 2010b), mutant strain AcR also outperformed wild-type strain ZM4 in MM supplemented with NaAc (Figure 1, Additional File 1). Strain AcR overcame the acetate stress and reached stationary phase after about 130 h post-inoculation while ZM4 reached stationary phase after approximately 166 h. Both strains achieved similar final cell densities based upon OD_600nm_ readings. Strain ZM4 had a longer lag phase and began to consume glucose much later than AcR, with most of the glucose in AcR cultures consumed at 148 h post-inoculation while cultures of ZM4 took 166 h to use the majority of the glucose in MM (Figure 1). As glucose was consumed earlier by AcR cultures, there was also a concomitant earlier onset for ethanol production compared to ZM4. Both strains produced similar yield of ethanol by the end of the experiment with acetate concentration kept steady during the whole experiment (Figure 1).

### *Z. mobilis* wild-type ZM4 and AcR mutant proteomic profiling differences in MM

Quantitative proteomics was used to compare the proteomic differences between *Z. mobilis* wild-type ZM4 and AcR mutant cells at 148 and 190 h post-inoculation. We identified 705 chromosomal encoded proteins and 6 plasmid derived proteins for the 148 h comparison, and 728 chromosomal proteins and 7 plasmid proteins for the 190 h comparison. We thus identified 638 chromosomal proteins and 4 plasmid proteins in common for the two time points. Altogether, this study identified 795 unique chromosomal proteins and 9 unique plasmid proteins, which is about 46% of the predicted *Z. mobilis* ZM4 proteome based upon a reannotation of the genome (Yang et al., 2009a) (Additional Files 2, 3A). The PI and MW distributions for the identified proteins were similar to that of the predicted theoretical PI and MW distributions for all proteins (Additional File 4). This study represents, to our knowledge, the largest quantitative measurement of the *Z. mobilis* proteome published to date.

The 148 h time point comparison between ZM4 and AcR strains revealed 120 proteins that had at least 1.5-fold differences in abundance and were significantly different based upon the ProRata likelihood algorithm for quantitative shotgun proteomics and a confidence interval of 90% as described previously (Pan et al., 2006). This included 55 upregulated proteins and 65 downregulated proteins in AcR compared to ZM4, of which 11 were upregulated and 25 were downregulated more than 2-fold (Additional File 2). There were 107 proteins with a significant difference and at least a 1.5-fold change in levels for stationary phase comparison (190 h) using the same criteria. In strain AcR, 47 proteins were upregulated and 60 downregulated compared to ZM4, of which 10 were upregulated and 26 were downregulated and had changes of at least 2-fold (Additional File 2). Approximately half of the proteins that were identified as being different for one time point were also statistically different in the other time point (Additional Files 2, 3B–C).

The interactions among the down-regulated or up-regulated proteins with at least a 1.5-fold change were also analyzed for their previously documented interactions using STRINGS database (Jensen et al., 2009). The 14 proteins consistently upregulated in AcR for both time points compared to ZM4 had fewer interactions (Additional Files 2, 3D). Among the 39 proteins downregulated in AcR at both the 148 and 190 h time points post-inoculation, stress-responsive proteins such as catalase (ZMO0918), glutathione synthetase (ZMO1913), glutaredoxin 2 (ZMO0070), and a glutaredoxin-related protein (ZMO1873) were downregulated and most of them have been shown to interact with one another (Additional Files 2, 3E). Other proteins downregulated and connected included preprotein translocase (ZMO1896-8), cofactor synthesis of biotin synthase (ZMO0094), and amino acid biosynthesis such as shikimate 5-dehydrogenase (AroB, ZMO0041), threonine synthase (ThrC, ZMO1891), and 3-isopropylmalate dehydratase large subunit (LeuC, ZMO0105) (Additional Files 2, 3E).

Although our intention was to identify differentially expressed proteins more confidently by combining datasets from both time points than merely the second time point of stationary at 190 h to exclude the growth effect, the first comparison however will be potentially problematical due to the growth difference between AcR mutant and wild-type ZM4 at 148 h time point (Figure 1). Therefore, although comparison for second time point of stationary phase at 190 h is reasonably accurate, caution should be taken to interpret proteomic comparison result of second time point due to potential growth effect impact. To overcome this problem, samples from same growth phase between AcR mutant and ZM4 wild-type were used for our transcriptomic comparisons as discussed below.

### *Z. mobilis* wild-type ZM4 and AcR mutant transcriptomic profiling differences in MM

Transcriptomic profiles for ZM4 and AcR in the presence of NaAc in MM were examined using NimbleGen high density expression arrays, essentially as reported previously (Yang et al., 2010b). The expression profiles generated in this study have been deposited in the GEO database (accession number GSE25443). About seventeen hundred genes were identified to be significantly differentially expressed using ANOVA modeling with strain (ZM4 and AcR) and time points post-inoculation as variables in MM (Additional File 5), which covered nearly all of the reannotated *Z. mobilis* ZM4 genes (Yang et al., 2009a). Hence dynamic gene expression changes were observed and for NaAc responses, 474 genes were significantly differentially expressed with at least a 2-fold change in expression values (Additional File 5).

Nine differentially expressed genes from different functional categories with a broad range of expression ratios were chosen for real-time quantitative PCR (RT-qPCR) validation (Additional File 6). RT-qPCR results indicated a high degree of concordance between microarray and RT-qPCR data with R-squared correlation coefficient values of 0.88, 0.87, 0.71, or 0.78 for the time points of 130, 148, 166, or 190 h respectively (Additional File 7).

Four genes were upregulated and 30 genes downregulated significantly with at least 2-fold changes in AcR when comparing AcR expression profiles to ZM4 profiles for all the time points (Additional File 5). Similar to the transcriptomics study in RM as reported previously (Yang et al., 2010b), the sodium/proton antiporter gene (ZMO0119) was also upregulated and ZMO0117 was downregulated in AcR in MM conditions. The other three genes that were consistently upregulated in AcR were L-asparaginase (ZMO1683), beta-fructofuranosidase (ZMO0375), and aldose 1-epimerase (ZMO0889). The 29 remaining genes that were consistently downregulated encoded mostly ribosomal proteins as well as proteins related to chemotaxis and flagellar biosynthesis, electron transport, fatty acid biosynthesis and protein export (Additional File 5).

### Correlations between proteomic and transcriptomic data of ZM4 and AcR in MM

**Figure 2:**
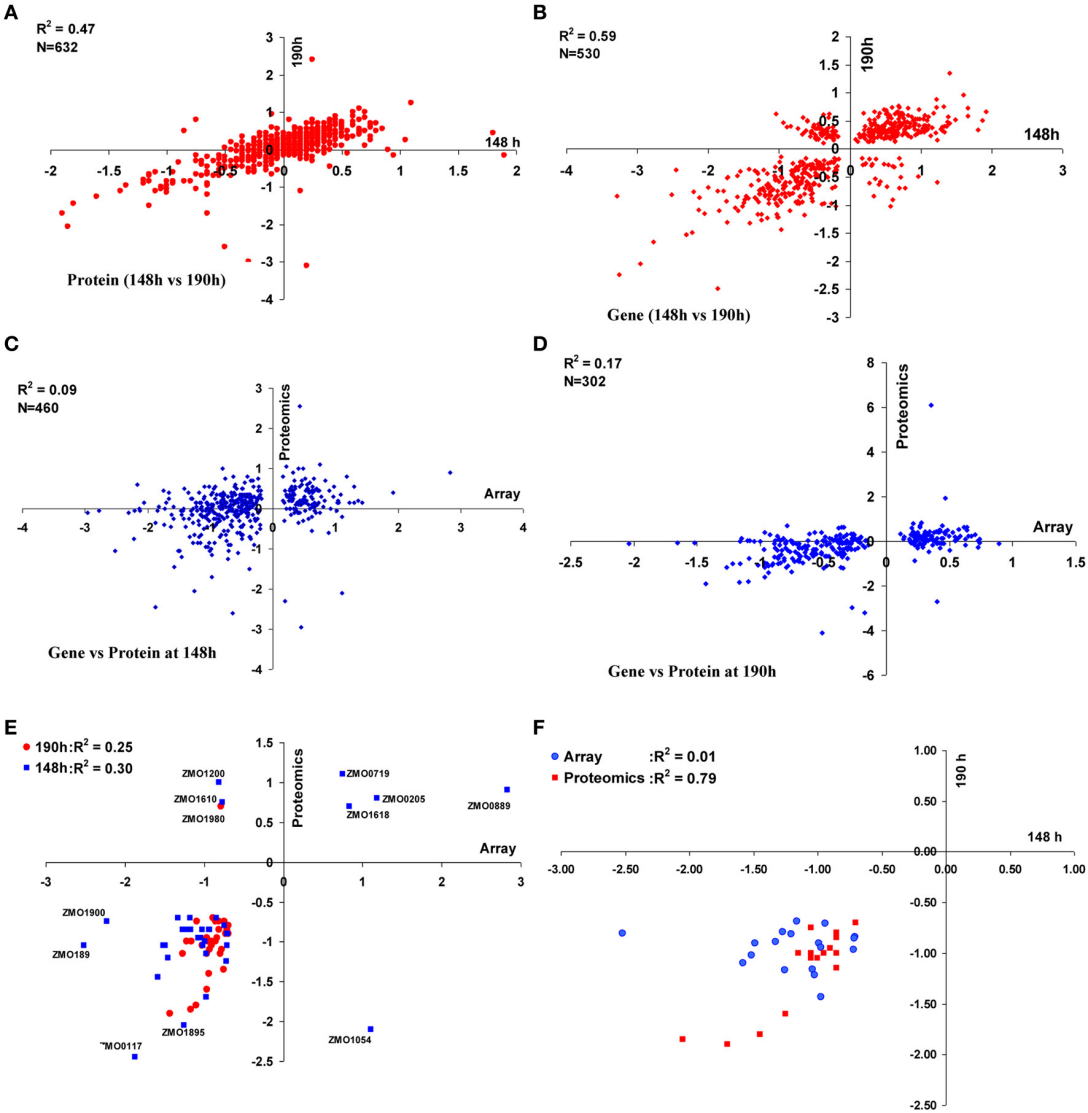
**Correlations between proteomic and transcriptomic data of ZM4 and AcR in MM**. Correlation within proteomics or transcriptomics shared at time point of 148 and 190 h between 632 proteins **(A)** or 530 genes **(B)** differentially expressed between AcR and ZM4 (AcR/ZM4) in MM; and the correlation between proteomics or transcriptomics at different time points of 148 h **(C)** or 190 h **(D)** post-inoculation; as well as correlations between significant gene-protein pairs with at least 1.5-fold changes between transcriptomics and proteomics **(E)**; and the correlation between common genes or proteins with at least 1.5-fold significant changes shared between the time point of 148 h and 190 h post-inoculation within transcriptomics or proteomics **(F)**. Numbers of X-axis and Y-axis are log_2_-based values.

We also examined the correlations between the gene or protein expression ratios at two time points of 148 and 190 h post-inoculation. There were 632 common proteins identified in both the 148 and 190 h time points. When the relative log_2_ ratios (AcR/ZM4) were compared, a moderate correlation (*R*^2^ = 0.47) was observed between the different sampling times (Figure 2A). Similarly, the correlation of transcriptomic profiles (AcR/ZM4) between 148 and 190 h was moderately high (*R*^2^ = 0.59) for the 530 common genes that showed statistically significant differential expression, but had not undergone any fold-change filtering (Figure 2B). When transcriptomic and proteomic data from the same time point (either 148 or 190 h) were compared, there are 460 gene-protein pairs for the 148 h time point that correlated poorly (*R*^2^ = 0.09) (Figure 2C), and the correlation between the 302 gene-protein pairs at 190 h post-inoculation is also relatively low (*R*^2^ = 0.17)(Figure 2D).

We have reported that the correlation between transcriptomic and proteomic data increases when only the significant genes or proteins were included for comparison, and the correlation even increases more when the ratios of transcriptomics or proteomics comparison increases from our recent *Z. mobilis* ethanol stress experiment of high-density microarray and shot-gun proteomics (Yang et al., 2013). Similarly, in this study, when only the genes or proteins with a significant and a ≥1.5-fold change were used for comparison purposes, the correlation between transcriptomic and proteomic data increased to *R*^2^ = 0.30 (*N* = 35) and *R*^2^ = 0.25 (*N* = 32) for 148 and 190 h comparisons, respectively (Figure 2E). Eighteen common gene-protein pairs were identified between proteomics and transcriptomics data in both 148 and 190 h post-inoculation with at least a 1.5-fold significant change, and all were downregulated in AcR (Table 1). The correlation for the proteomic study between 148 and 190 h went up to *R*^2^ = 0.79 (*N* = 18) when significance and fold change were considered (Table 1, Figure 2F). However, the correlation for the transcriptomic study between 148 and 190 h were decreased to 0.01 (*N* = 18) when significance and fold change criteria were applied (Table 1, Figure 2F). This result may suggest that change at transcriptional level is rapid which is consistent with our transcriptomic study in *Clostridium thermocellum* that genes were differentially expression even after several minutes post ethanol or furfural shock (Yang et al., 2012; Wilson et al., 2013). Moreover, this study also suggested that the change at protein level is relatively steady with potential post-transcriptional modification existing.

**Table 1:** **Gene-protein pairs identified in both transcriptomic and proteomic studies at both 148 and 190 h post-inoculation, and those only identified in either 148 or 190 h post-inoculation**.

**Gene**	**Product**	**A148**	**P148**	**A190**	**P190**
**18 TRANSCRIPTOMICS-PROTEOMICS PAIRS IDENTIFIED IN BOTH 148 AND 190 h**					
ZMO0009	Sulfite reductase [NADPH] flavoprotein, alpha chain	−1.20	−0.85	−0.81	−0.85
ZMO0020	Protein of unknown function DUF162	−1.58	−1.45	−1.10	−1.80
ZMO0021	Protein of unknown function DUF162	−0.97	−1.70	−1.43	−1.90
ZMO0027	IMP cyclohydrolase	−1.52	−1.05	−1.02	−1.05
ZMO0035	Ankyrin	−0.72	−1.25	−0.96	−1.60
ZMO0041	Shikimate 5−dehydrogenase	−0.71	−0.90	−0.84	−0.95
ZMO0079	Response regulator receiver protein	−0.71	−1.05	−0.85	−1.00
ZMO0090	Short−chain dehydrogenase/reductase SDR	−0.99	−1.00	−0.90	−1.05
ZMO1372	Short−chain dehydrogenase/reductase SDR	−1.27	−0.85	−0.79	−1.15
ZMO1891	Threonine synthase	−1.04	−0.95	−1.16	−1.00
ZMO1895	PfkB domain protein	−1.26	−2.05	−1.17	−1.85
ZMO1896	Protein−export membrane protein SecF	−1.33	−0.70	−0.89	−0.70
ZMO1897	Protein−export membrane protein SecD	−2.52	−1.05	−0.81	−0.75
ZMO1912	GTP−binding protein YchF	−0.97	−1.15	−0.95	−1.00
ZMO1913	Glutathione synthetase	−1.16	−0.85	−0.69	−0.80
ZMO1927	Signal peptide peptidase SppA, 67K type	−1.49	−1.05	−0.90	−1.00
ZMO1955	Malate dehydrogenase	−0.94	−0.85	−0.71	−0.85
ZMO1964	Glutamyl−tRNA synthetase	−1.02	−0.85	−1.22	−1.00
**Gene**	**Product**	**A148**	**P148**		
**17 UNIQUE TRANSCRIPTOMICS-PROTEOMICS PAIRS IDENTIFIED ONLY IN 148 h**					
ZMO1905	Glycerol−3−phosphate dehydrogenase	−0.93	−0.85		
ZMO1900	Fatty acid/phospholipid synthesis protein PlsX	−2.22	−0.75		
ZMO1899	3−oxoacyl−(acyl−carrier−protein) synthase III	−1.46	−1.20		
ZMO1898	Preprotein translocase, YajC subunit	−1.07	−0.95		
ZMO1875	Protein of unknown function DUF1476	−0.84	−0.70		
ZMO1618	Carbamoyl−phosphate synthase, small subunit	0.84	0.70		
ZMO1610	Hypothetical protein	−0.77	0.75		
ZMO1200	Sugar−phosphate isomerase, RpiB/LacA/LacB family	−0.81	1.00		
ZMO1054	DNA topoisomerase IV, A subunit	1.10	−2.10		
ZMO0889	Aldose 1−epimerase	2.83	0.90		
ZMO0719	Lytic transglycosylase catalytic	0.75	1.10		
ZMO0205	NAD−dependent epimerase/dehydratase	1.18	0.80		
**ZMO0117**	**hybrid cluster protein**	**−1.87**	**−2.45**		
ZMO0094	Biotin synthase	−1.18	−0.70		
ZMO0080	CheD, stimulates methylation of MCP proteins	−0.70	−0.90		
ZMO0022	Protein of unknown function	−1.01	−1.05		
ZMO0013	Purine NTP pyrophosphatase, rdgB/HAM1 family	−0.74	−0.80		
**Gene**	**Product**	**A190**	**P190**		
**14 UNIQUE TRANSCRIPTOMICS−PROTEOMICS PAIRS IDENTIFIED ONLY IN 190 h**					
ZMO0017	Fmu	−0.73	−0.90		
ZMO0056	Glucosamine/fructose−6−phosphate aminotransferase	−0.78	−1.10		
ZMO0062	Aldo/keto reductase	−0.97	−0.95		
ZMO0070	Glutaredoxin, GrxB family	−0.75	−1.35		
ZMO0106	3−isopropylmalate dehydratase, small subunit	−1.09	−0.75		
ZMO1885	NADH:flavin oxidoreductase/NADH oxidase	−0.74	−0.75		
ZMO1956	DNA repair protein RecN	−0.81	−0.75		
ZMO1963	Citrate synthase I	−0.86	−0.75		
ZMO1970	3−methyl−2−oxobutanoate hydroxymethyltransferase	−0.69	−0.90		
ZMO1980	Methyltransferase GidB	−0.78	0.70		
ZMO1984	Aldo/keto reductase	−0.96	−1.15		
ZMO1989	Methylated−DNA/protein−cysteine methyltransferase	−0.93	−1.10		
ZMO1992	Carboxymethylenebutenolidase	−0.94	−1.40		
ZMO1993	Alcohol dehydrogenase GroES domain protein	−1.27	−1.15		

*A148 and A190: array result comparing AcR to ZM4 at time point 148 and 190 h post-inoculation respectively; P148 and P190: proteomics result comparing AcR to ZM4 at time point 148 and 190 h post-inoculation respectively. All the gene-protein pairs are statistically significant with at least 1.5-fold difference between AcR and ZM4 in both transcriptomic and proteomic studies. Negative number indicates gene/protein was downregulated comparing AcR to ZM4 and positive number indicates upregulation*.

### Metabolic pathway analyses of proteomic and transcriptomic data of ZM4 and AcR in MM

The PathwayTools Omics Viewer (Karp et al., 2002, 2011) was used to further examine transcriptomics and proteomics data and their relationships. Genes in the fatty acid and hopanoid biosynthetic pathways are upregulated in ZM4 compared to AcR (Additional File 8A). Hopanoids have been found in a variety of bacteria including *Z. mobilis* and are reported to protect against the toxic effects of ethanol (Flesch and Rohmer, 1989; Hermans et al., 1991; Horbach et al., 1991; Shigeri et al., 1991; Welander et al., 2009). For example, they have also been reported to play a role in *Rhodopseudomonas palustris* membrane integrity and pH homeostasis (Welander et al., 2009). However, there is debate of their role in *Z. mobilis* (Moreau et al., 1997) and we didn't identify obvious differential gene expression when *Z. mobilis* encountered ethanol stress (Yang et al., 2013). Further study is required to elucidate their role in *Z. mobilis*, and their relative contribution, if any, to sodium acetate sensitivity or tolerance to the strains used in this experiment.

In other pathway comparisons of AcR to ZM4 at 148 and 190 h, the strains had similar patterns of protein detection, with 120 or 107 differentially expressed proteins with ≥1.5-fold change at each time point respectively were used for analyses (Additional Files 8B,C). We identified 509 chromosomal gene/protein pairs that have no significant changes between AcR and ZM4 in MM with NaAc in both 148 and 190 h post-inoculation (Additional File 2), the majority of which are involved in the ED and mixed-acid fermentation pathways, and tRNA charging (Additional File 8A–C). When the log_2_ transformed peptide hits of all the proteins identified in 148 and 190 h post-inoculation were used for pathways analyses, proteins involved in ED pathway and ethanol production are among the most abundant proteins (Additional Files 2, 8D). The abundance of ED pathway and ethanol production enzymes and the relatively stability of protein levels between ZM4 and AcR indicates that this pathway for carbon and electron flow are core elements in the physiology of both strains.

Recently, a number of studies have added important details to our understanding of *Z. mobilis* physiology (Kouvelis et al., 2009; Yang et al., 2009a, 2010a,b; Kerr et al., 2010; Linger et al., 2010; Zhang et al., 2010; Widiastuti et al., 2011). However, many genes encode proteins for which we now have proteomics evidence of their expression, their functions remain unknown. The largest quantitative measurement of the *Z. mobilis* proteome obtained in this study may help elucidate *Z. mobilis* genes, proteins, their functions and regulation.

### ZM4 and AcR transcriptomic profiles in different conditions

**Figure 3:**
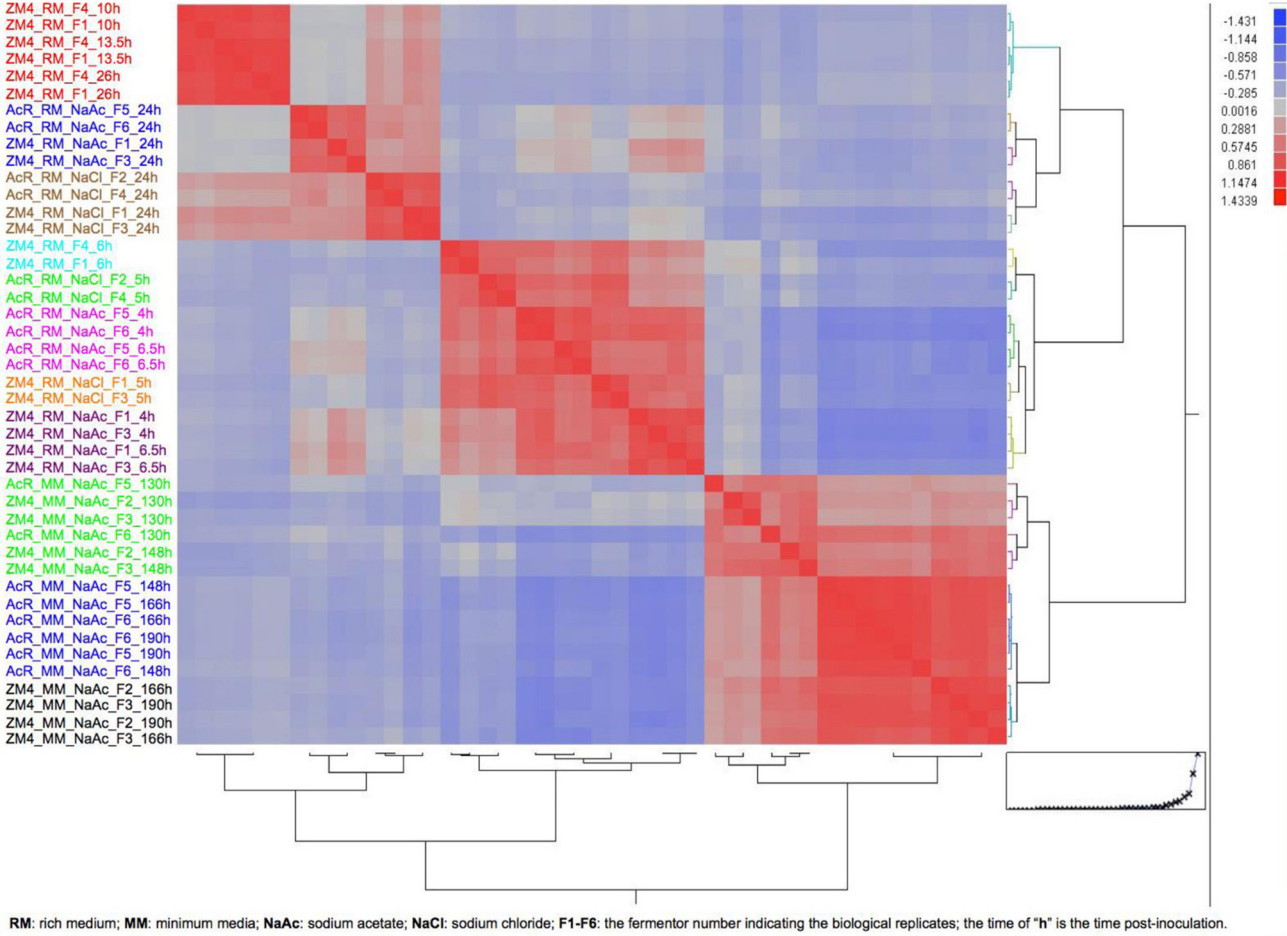
**The heat map and dendrogram of hierarchical clustering analysis on correlation among 44 arrays for growth phase determination**. The information about each array is listed on figure left and the color from top to bottom indicates different growth media and phase.

In this study, we also combined *Z. mobilis* microarray data collected from previous studies with expression data collected in this experiment to provide greater insights in *Z. mobilis* physiology and gene regulation. Forty-four microarrays were used in the analysis (Additional File 9) to compare the gene expression differences between the variables of strain (AcR and ZM4), growth phase (exponential and stationary), media (RM and MM), and treatment (NaCl, NaAc, and control of RM only). Considering the growth differences between ZM4 and AcR in this experiment (Figure 1) and the fact that *Z. mobilis* has dramatic transcriptional differences between exponential and stationary phase (Additional Files 5, 10) (Yang et al., 2009b), the relative large number of genes significantly differentially expressed at time point 148 h post-inoculation between AcR and ZM4 as well as the low transcriptomic correlation between 148 and 190 h post-inoculation is likely associated with the growth phase differences. To ensure compatible comparisons were being made among different time points in RM or MM under different conditions, the correlations among all the forty-four microarrays were investigated using the JMP Genomics 4.0 (SAS, NC) and based upon a hierarchical clustering and growth curve analyses (Figure 3), time points were assigned to either exponential or stationary phase for further statistical analyses (Figure 3, Additional File 9). Differences between the variables of strain, growth phase, media, and treatment were then analyzed by ANOVA, as described previously (Yang et al., 2009b, 2010b). The ANOVA identified that nearly every gene in the *Z. mobilis* genome was differentially expressed under one or more of the many conditions tested in this global analysis (Additional File 11), leading to an opportunity to identify the strain, media, growth phase or treatment responsive genes, as discussed below.

#### Strain-specific genes

Gene expression profiles for ZM4 and AcR were compared and when all the conditions (Additional File 9) were taken into account, only five genes were significantly differentially expressed with at a least 2-fold change between strains. The significant differentially expressed strain specific genes included two genes with increased expression and three genes downregulated in strain AcR (Additional Files 11, 12). We have previously reported that *nhaA* (ZMO0119) is upregulated and ZMO0117 is downregulated in AcR compared to ZM4 in exponential and stationary growth conditions for RM (Yang et al., 2010b). In this study, which considered more conditions, we identified that that another gene (hypothetical protein ZMO1787, 82aa) was consistently upregulated in AcR under the conditions tested and two AcR genes encoding a predicted permease (ZMO0055, 263 aa) and a conserved hypothetical protein (ZMO0025, 234 aa) were consistently downregulated Additional File 11).

#### Media-responsive genes

Gene expression differences between MM and RM in the presence of NaAc were compared (Additional File 9). When the strain responses of both AcR and ZM4 in both exponential and stationary phases were combined to examine media differences, 232 genes were identified as being upregulated and 247 downregulated in MM compared to RM (Additional File 13). Almost half of upregulated genes encode hypothetical proteins with unknown functions, and many of the remaining upregulated genes encode proteins involved in stress sensing and responses. These included two-component signal transduction genes (7), transcriptional regulators (11), sigma factors (*rpoD*, *rpoH*), flagellar and chemotaxis genes, and other stress responsive genes such as an putative operon containing heat-inducible transcription repressor *hrcA* (ZMO0015) and chaperone gene *grpE* (ZMO0016). Other genes included cold shock gene (ZMO0925), DnaJ-class chaperone (ZMO1069) and chaperone *hspD* (ZMO0989), three glutaredoxin-related genes, three thioredoxin-like genes, and genes associated with DNA repair such as UvrABC system gene *uvrB* (ZMO0362), *mutM* (ZMO1187), *mutS* (ZMO1907) and ZMO1426 (Additional File 13).

There are more previously documented interactions among the downregulated genes in MM relative to RM compared to the upregulated genes (Additional File 14). These MM-downregulated genes are mostly related to the central carbon metabolism, which included genes such as 6-phosphogluconolactonase gene *pgl* (ZMO1478), phosphoglycerate mutase gene *pgmA* (ZMO1240), gluconolactonase gene *gnl* (ZMO1649), pyruvate decarboxylase gene *pdC* (ZMO1360), and pyruvate dehydrogenase gene *pdhA* and *pdhB* (ZMO1606, ZMO1605) (Additional Files 13, 14B). Genes involved in amino acid biosynthesis, encoding ribosomal proteins and genes for nucleotide biosynthesis, as well as genes related to energy metabolism such as electron transport system and ATP generation were also mainly down-regulated in MM (Additional Files 13,14B).

#### Treatment-responsive genes

The following conditions were used to examine treatment-specific responses: ZM4 grown in RM; both ZM4 and AcR growth in RM with NaCl, RM with NaAc, or MM with NaAc (Additional File 9). These conditions were used to make the following comparisons: treatment of NaCl or NaAc in RM vs. RM for strain ZM4; and as the treatment of NaAc vs. NaCl in RM for both ZM4 and AcR strains (Additional File 13).

The NaCl-responsive genes in the ZM4 wild-type background included 88 downregulated genes and 47 upregulated genes (Additional Files 13, 15A,B). More genes were responsive in a ZM4 background grown in RM supplemented with NaAc, including 159 downregulated and 103 upregulated genes. This was approximately twice the number as responsive to the less severe NaCl treatment (Additional Files 13, 15C,D). Differentially expressed genes shared between NaCl and NaAc in RM compared to RM included 27 upregulated and 66 downregulated genes (Additional Files 13, 15E,F). Genes upregulated in both NaCl and NaAc treatment related to cysteine synthesis, with 6 genes clustered together (*cysD, I, J, K, N*, ZMO0006 and ZMO0055) and tryptophan synthesis (*trpB, F*), and most of the downregulated genes encoded hypothetical proteins with unknown function with the exception of several related to flagellar biosynthesis and sucrose metabolism (Additional Files 13, 15).

When the responses of *Z. mobilis* in RM with NaAc were compared to that of NaCl, there were 37 upregulated and 41 downregulated genes (Additional Files 13, 15I,J). Genes related to ribosomal proteins and amino acid biosynthesis were downregulated and upregulated genes related to the Entner–Doudoroff (ED) pathway (ZMO1518 and *pgi*), energy metabolism [e.g., electron transport (ZMO0021, ZMO1851, ZMO1885) and ATP synthesis gene *atpC* (ZMO0242)], cell wall formation (ZMO1724), regulator gene *zrp* (ZMO0372), and several transporter related genes such as signal peptidase I gene *lepB* (ZMO1710), Sec-independent protein translocase tatA/E homolog gene (ZMO1220), TolQ biopolymer transport gene (ZMO0161) and biopolymer transport gene *exbD* (ZMO1715), Fe^2+^ transport system gene *feoB* (ZMO1541), and potassium transport system gene *kup* (ZMO1209).

#### Growth phase-dependent genes

When strain profiles in the presence of NaAc were considered together and a ≥2-fold change was applied, 331 genes were significantly differentially expressed in a phase-dependent manner for RM (Additional Files 11, 12). When the RM NaCl profiles were analyzed, 661 genes were identified (Additional Files 11, 12). Similar patterns were identified for the comparisons when variable of strain was taken into consideration separately (Additional Files 11, 12). In the presence of NaAc, the stationary phase upregulated genes were less correlated than the downregulated ones (Additional Files 16). The genes downregulated in stationary phase are related to ribosomal proteins, chemotaxis and flagellar systems, amino acid and nucleotide biosynthesis, and electron transport (Additional Files 11, 16A). Except for those encoding hypothetical proteins, genes upregulated in stationary phase are related to stress responses such as the catalase gene (ZMO0918), glutaredoxin gene (ZMO0070), thioredoxin gene (ZMO1705), ferredoxin gene *fdxN* (ZMO1818), organic hydroperoxide resistance gene (ZMO0693), ATP-dependent Clp protease gene *clpB* (ZMO1424), RNA polymerase sigma-32 factor gene *rpoH* (ZMO0749), and integration host factor gene *ihfB* (ZMO1801) (**Additional Files 11, 16B**).

## Discussion

### Genes contributing to sodium acetate tolerance phenotype of AcR

AcR mutant had advantages under NaAc stress in both RM (Yang et al., 2010b) and MM conditions, with shorter lag phase leading to earlier glucose consumption and ethanol production than ZM4 cells (Figure 1). We have reported previously that the truncation within gene ZMO0117 in AcR causes the consistent upregulation of the sodium proton antiporter gene *nhaA* (ZMO0119), which is the determining factor of AcR mutant for sodium acetate tolerance in RM (Yang et al., 2010b). In this study, the *nhaA* gene also had higher expression levels in strain AcR compared to ZM4 in MM (Additional File 11). ZM4 *nhaA* expression in unamended RM was higher (~2-fold) in stationary phase compared to exponential phase (Additional File 11) and differential expression was also measured for other conditions and strain comparisons (Additional File 13). Acetate accumulates in stationary phase *Z. mobilis* fermentations (Yang et al., 2009b) and greater expression of *nhaA* gene may ameliorate cellular function and activity under such stressful conditions.

In addition, compared to ZM4, the ZMO0117 gene in AcR showed a similar profile of down-regulation in MM (Additional File 11) to that of in RM (Yang et al., 2010b). Proteomic data also showed that the peptide hits for ZMO0117 were less abundant in AcR compared to ZM4 at 148 h post-inoculation (Additional File 2). However, the NhaA protein was not detected in any sample in this experiment (Additional File 2) and yet differential expression was observed for this gene, which demonstrates the value of conducting integrated experiments and may point to a technical challenge in detecting hydrophobic proteins like NhaA that have many transmembrane spanning domains (Poetsch and Wolters, 2008; Gilmore and Washburn, 2010).

As discussed above, this study also identified other AcR specific genes. For example, ZMO0055 was significantly downregulated in AcR compared to ZM4 in all conditions with NaAc and responded to other conditions too (Additional File 11). ZMO0055 is predicted to belong to the sulfite exporter TauE/SafE family protein (pfam01925), a family of integral membrane proteins involved in transporting anions across the cytoplasmic membrane during taurine metabolism as an exporter of sulfoacetate (Weinitschke et al., 2007). As discussed above, ZMO0055 is closely related to CysC, D, I, N (ZMO0003, 4, 5, 8) based on protein analysis using String pre-computed database (Additional File 15), which were upregulated in both NaCl and NaAc treatment. In addition, recent work by Skerker et al. (2013) also indicated that cysteine synthases, which are required for L-cysteine biosynthesis from sulfate, are related to hydrolysate tolerance. They proposed that the increased demand for sulfite and cysteine is due to the increased needs of glutathione, which is formed from glutamate and cysteine, during *Z. mobilis* growth in stressful condition (Skerker et al., 2013). Therefore, the downregulation of sulfite exporter potentially could help Z. *mobilis* retain sulfite for enhanced sodium acetate tolerance phenotype. However, further detailed study is needed to understand the role and contribution of these AcR specific genes, which will help reveal the complete difference between AcR and ZM4 and at the same time help assign the function for these AcR-specific genes with hypothetical function (ZMO1787 and ZMO0025).

### Relationship among media, inhibitor, growth phase, and gene expression dynamics

The gene expression intensity mean value of the significantly expressed genes (Additional File 17) were further used for hierarchical clustering to compare each gene at 14 different conditions, the result indicated that minimum medium had the most dramatic effect on gene expression compared to those in RM, followed by growth phase, inhibitors (NaAc>NaCl), and strain (Additional File 18A). Gene expression profiles in MM were clustered together and separated from the rest in RM (Additional File 18A). *Z. mobilis* growth in MM has a longer lag phase and generates a lower final cell density compared to that in RM (Additional File 1) (Yang et al., 2010b), which indicates that one or more factors are limiting *Z. mobilis* growth in MM, and MM is possibly a more stressful environment for *Z. mobilis* cells which is further supported by the upregulation of stress-responsive genes in MM compared to that in RM (Additional Files 13, 14).

The second level of clusters was between exponential and stationary growth phases, consistent with an earlier global profiling report for strain ZM4 that growth phase tends to have more dramatic effect on gene expression than a single stressor (Yang et al., 2009b). The greatest number of differentially expressed genes between exponential and stationary phases is observed for strain ZM in RM, followed by strain ZM in RM with NaCl, RM with NaAc and then ZM4 in MM (Additional File 12). In RM, *Z. mobilis* growth is inhibited by NaCl but NaAc is more inhibitory (Yang et al., 2010b), and a similar trend was observed in MM (Additional File 1). In this experiment, the inhibitor (NaAc) was added into the medium before the fermentation allowing the strains to respond and adapt to the conditions for several generations before samples were taken for systems biology studies. Hence, when transcriptomic and proteomic profiles between strains are compared under the same growth condition, the differences likely represent differences in homeostasis that allow AcR to function better than ZM4. The two strains were in different growth phases for the first proteomics time point, which confounds strain comparisons for tolerance but does still allow gene-protein relationships to be examined. In general, the large datasets reported in this study will allow others to investigate aspects such as codon bias and RNA secondary structures.

In addition, the microarray results for each gene at different conditions can help us understand the microbial physiology and identify consistently strong or inducible promoters for metabolic engineering application. For example, in this study, 52 genes and 6 other genetic features were among the top 2.5% of all genetic features with consistently strong expression intensity. Except for a dozen of genes encoding hypothetical proteins, they involve in protein biosynthesis, glycolysis and fermentation as well as ATP synthesis and stress response indicating their key role on *Z. mobilis* metabolism (Additional File 17). Several papers have been published recently to study the stress responses of *Z. mobilis* to different inhibitors (e.g., ethanol, furfural) using systems biology approaches (Yang et al., 2009b, 2010b, 2013; He et al., 2012a,b; Jeon et al., 2012; Skerker et al., 2013), we are currently working on several transcriptomics studies using microarray and next-generation sequencing based strand-specific RNA-Seq technique. Combining all these systems biology datasets, we will revisit this topic to investigate the impact of different variables of media, carbon source (glucose or xylose), different inhibitors, and growth phase on *Z. mobilis*. The transcriptional profiles will be further compared to help identify the condition specific promoters more confidently, and the discrepancy between transcriptomics and proteomics at different conditions will help understand the post-transcriptional regulation mechanism.

## Conclusions

Our study has provided the global profiling of the model ethanogenic bacterium *Z. mobilis* at both transcriptomic and proteomic levels in minimal medium (MM) for the first time. The results indicated that AcR had similar advantage in MM as in RM. AcR mutant can overcame acetate stress earlier with shorter lag phase, earlier glucose utilization and ethanol production than wild-type ZM4, and sodium proton antiporter gene (*nhaA*) also plays an important role in sodium acetate tolerance in MM as that of in RM we reported previously (Yang et al., 2010b). In addition, this is also the first attempt that we are aware of to combine massive transcriptomic data for condition-specific gene identification. The proteomic and transcriptomic data generated in this study and the one we have reported will provide massive datasets for future metabolic modeling and strain improvement.

## Materials and methods

### Strains and growth conditions

Wild-type *Z. mobilis* ZM4 was obtained from the American Type Culture Collection (ATCC 31821). *Z. mobilis* acetate tolerant strain AcR has been described previously (Joachimstahl et al., 1998). ZM4 and AcR were cultured in RM (Glucose, 20.0 g/L; Yeast Extract, 10.0 g/L; KH_2_PO_4_, 2.0 g/L, pH5.0) (Yang et al., 2009b) for routine strain maintenance. MM was similar to the one reported to isolate auxotrophic *Z. mobilis* mutants (Goodman et al., 1982): 20 g Glucose, 1 g KH_2_PO_4_, 1 g K_2_HPO_4_, 0.5 g NaCl, 1 g (NH_4_)_2_SO_4_ was dissolved in 986.5 mL distilled H_2_O with pH adjusted to pH 5.0 before autoclave. The sterilized MM broth was then cooled to below 55°C before 500 mg MgSO_4_.7H_2_O (2.5 mL 200 g/L stock), 25 mg Na_2_MoO_4_.2H_2_O (1 mL 25 g/L stock), and 10 mL Vitamin (ATCC MD-VS) were added to make a 1-L MM.

Growth assays were used to identify the effects of sodium acetate (NaAc) and sodium chloride (NaCl) on the growth of *Z. mobilis* in MM for subsequent fermentation experiments using a Bioscreen C instrument (GrowthCurves USA, NJ). Fermentations were conducted in 7.5-L BioFlo110 bioreactors (New Brunswick Scientific, NJ) fitted with agitation, pH, and temperature probes and controls, and bacterial growth was monitored turbidometrically by measuring optical density at 600_nm_ with a model 8453 spectrophotometer (Hewlett-Packard, CA.) as described previously (Yang et al., 2009b), except that the fermentation volume was 4 L. Samples were harvested during fermentation at different time points (Figure 1) as described previously (Yang et al., 2009b).

### HPLC

HPLC analysis was used for the measurements of the concentration of glucose, acetate, and ethanol in 0.2 μm-filtered samples taken at different time points during fermentation (Figure 1) and analyzed as described previously (Yang et al., 2009b).

### Proteome sample preparation

Duplicate mixtures of microbial cells that were metabolically labeled with either ^15^N ammonium sulfate for the wild-type *Z. mobilis* ZM4 culture or ^14^N ammonium sulfate for the AcR mutant culture were prepared by mixing equal weights of cell pellets from duplicate cultures for each strain. Cell mixtures were lysed by sonication in ice-cold 50 mM Tris-HCl (pH 7.5) buffer, and unbroken cells were removed by centrifugation at 5000 × g for 10 min. Protein concentration for each sample was determined with the RC DC™ protein assay (Bio-Rad Lab, CA). The two fractions from each cell mixture were digested using the following protocol. The proteins were denatured and reduced with 6 M guanidine and 10 mM dithiothreitol (DTT) (Sigma-Aldrich, MO) at 60°C for 1 h. The denatured proteome fractions were diluted 6-fold with 50 mM Tris/10 mM CaCl_2_ (pH 7.6), and sequencing grade trypsin was added at the ratio of 1:100 (wt:wt). The first digestion was run overnight at 37°C and, after adding additional trypsin, the second digestion was run for 5 h at 37°C. The samples were then reduced with 20 mM DTT for 1 h at 60°C and were desalted using Sep-Pak Plus C-18 solid-phase extraction (Waters Co, MA).

### Quantitative proteomics measurement

The protein digests were examined with LC-MS/MS using twelve-step split-phase MudPIT (MacCoss et al., 2002; McDonald et al., 2002) in duplicate. The samples were loaded via a pressure cell (New Objective, MA) onto a 250-*u*m-I.D. fused silica front column fritted into an M-520 filter union (Upchurch Scientific, WA). The column packing consisted of 2 cm strong cation exchange resin Luna® and 2 cm C18 reverse-phase resin Aqua (Phenomonex, CA). A 100-*u*m-I.D. PicoFrit column (New Objective, MA) was packed with 15 cm C18 reverse-phase resin. The front column was connected with the PicoFrit column and then placed in-line with a Dionex Ultimate quaternary HPLC. Two-dimensional LC separation was performed with twelve salt pulses, each of which was followed by a 2-h reverse-phase gradient. MS/MS analysis was performed on an LTQ linear ion trap instrument (ThermoFinnigan, CA) with dynamic exclusion enabled. Each full scan (400–1700 *m/z*) was followed by three data-dependent MS/MS scans at 35% normalized collision energy. The full scans were averaged from five microscans and the MS/MS scans were averaged from two microscans.

### Quantitative proteomics data analysis

All MS/MS scans were searched in two iterations against the FASTA database containing all annotated *Z. mobilis* proteins using the SEQUEST program (Eng et al., 1994). In the first iteration, the molecular masses of amino acids containing ^14^N were used, and, in the second iteration, the masses of amino acids containing ^15^N were used. The peptide identifications from the two iterations were merged. The DTASelect program (Tabb et al., 2002) was used to filter the peptide identifications and to assemble the peptides into proteins using the following parameters: retaining the duplicate MS/MS spectra for each peptide sequence (DTASelect option: -t 0), fully tryptic peptides only, with a delCN of at least 0.08 and cross-correlation scores (Xcorrs) of at least 1.8 (for parent ion charge state, *z* = +1), 2.5 (*z* = +2), or 3.5 (*z* = +3). Selected ion chromatogram extraction, peptide abundance ratio estimation and protein abundance ratio estimation were completed with the ProRata program as described previously (Pan et al., 2006, 2008).

### Microarray analysis and qRT-PCR validation

Microarray analysis was conducted essentially as described previously (Yang et al., 2010b). Briefly, total cellular RNA was extracted using the TRIzol reagent (Invitrogen, CA) followed by RNase-free DNase I (Ambion, TX) digestion. RNA quality and quantity were tested with a NanoDrop ND-1000 spectrophotometer (NanoDrop Technologies, DE) and Agilent Bioanalyzer (Agilent, CA) before ds-cDNA synthesis using Invitrogen ds-cDNA synthesis kit (Invitrogen, CA). The ds-cDNA was sent to NimbleGen for labeling, hybridization, and scanning following company's protocols. Quality assessments, normalization, and statistical analyses were conducted using JMP Genomics 4.0 software (SAS Institute, Cary, NC) as described earlier (Yang et al., 2010b). An analysis of variance (ANOVA) determined differential expression levels between strains and time points using the FDR testing method (*p* < 0.05). The interaction among differentially-regulated genes was investigated using the String 8.2 database (Jensen et al., 2009), available at http://string.embl.de/. The transcriptomic and proteomic data are also mapped to predicted metabolic pathway using PathwayTools Omics Viewer (Karp et al., 2002, 2011) at http://biocyc.org/expression.html.

Microarray data were validated using real-time qPCR as described previously (Yang et al., 2009b, 2010b), except that the Bio-Rad MyiQ2 Two-Color Real-Time PCR Detection System (Bio-Rad Lab, CA) and Roche FastStart SYBR Green Master (Roche Applied Science, IN) were used for this study. Nine genes representing different functional categories and a range of gene expression values based on microarray hybridizations were analyzed using qPCR from cDNA derived from different time point samples. Primer pairs were designed as described previously (Yang et al., 2009b), and the oligonucleotide sequences of the nine genes selected for qPCR analysis are listed in Additional File 6. The qRT-PCR ratios were plotted against the microarray ratios and a regression analysis was conducted to generate R-squared correlation coefficient value.

## Author contributions

Steven D. Brown, Shihui Yang, Gregory B. Hurst, and Chongle Pan designed the experiment. Shihui Yang carried out the fermentation, RNA extraction, and sample preparation for HPLC, microarray, and proteomics. Lezlee Dice performed the qRT-PCR. Chongle Pan performed the proteomic runs and generated proteomic raw data. Shihui Yang, Steven D. Brown, Chongle Pan, and Gregory B. Hurst analyzed the data. Shihui Yang and Steven D. Brown wrote the manuscript, and Brian H. Davison, Gregory B. Hurst, and Chongle Pan provided inputs on manuscript revision.

### Conflict of interest statement

The authors declare that the research was conducted in the absence of any commercial or financial relationships that could be construed as a potential conflict of interest.
