# Supplementary material for: Elucidation of Zymomonas mobilis physiology and stress responses by quantitative proteomics and transcriptomics
Source: Front Microbiol. 2014 May 22;5:246. doi: 10.3389/fmicb.2014.00246 (PMC4033097; doi:10.3389/fmicb.2014.00246)

**Additional files**

Additional file 1

File format: DOC

Title: The effect of different NaCl or NaAc concentrations on *Z. mobilis* growth in MM.

Description: The growth of *Zymomonas mobilis* ZM4 was monitored by a Bioscreen C (Growth Curves USA, NJ) at 30^o^C under anaerobic conditions in an anaerobic growth chamber in minimum media (MM) broth with or without the supplementation of 12 or 16 g/L sodium acetate (NaAc) or same molar concentration of NaCl as that of NaAc at 8.65 or 11.4 g/L respectively.

Additional file 2

File format: EXCEL

Title: Proteins with peptides identified at time point of 148 h (**A**) and 190 h (**B**) post-inoculation and the difference between acetate tolerant mutant AcR and wild-type ZM4 (AcR/ZM4).

Description: The ratio is log2 based value.

Additional file 3

File format: DOC

Title: Correlation between the proteomic differences between strains AcR and ZM4, and the pre-existing interaction among common genes at time points of 148 h or 190 h post-inoculation.

Description: Venn diagrams of all the proteins (**A**); common upregulated ones (**B**); and common downregulated ones (**C**) with peptide identified at time points of 148 h or 190 h post-inoculation. And the interactions among the39 common upregulated (**D**) and 14 common down-regulated ones (**E**) in AcR at 148 and 190 h post-inoculation using the pre-documented interactions based on String 8.2 database. Greater numbers of lines are associated with increased connections and greater confident for associations. The network nodes are proteins. The edges represent the predicted functional associations. An edge may be drawn with up to 7 differently colored lines - these lines represent the existence of the seven types of evidence used in predicting the associations. A red line indicates the presence of fusion evidence; a green line - neighborhood evidence; a blue line - coocurrence evidence; a purple line - experimental evidence; a yellow line - textmining evidence; a light blue line - database evidence; a black line - coexpression evidence.

Additional file 4

File format: DOC

Title: Molecular weight (MW) and pI distribution of proteins identified through proteomics.

Description: Comparison the MW and pI distributions of 800 proteins (ca 46% of the total predicted proteins) identified from proteomics and the theoretical distribution based on genome prediction.

Additional file 5

File format: EXCEL

Title: Transcriptomic profiling of *Z. mobilis* acetate mutant AcR and wild-type ZM4 in the presence of NaAc in MM.

Description: Transcriptomic profiling of *Z. mobilis* acetate mutant AcR and wild-type ZM4 in the presence of NaAc in MM at time points of 130, 148, 166, and 190 h post-inoculation. The ratio is log2 based value.

Additional file 6

File format: DOC

Title: qPCR primers used in this study.

Description: The information about the primers used for qPCR to verify the microarray results.

Additional file 7

File format: DOC

Title: The correlation between microarray and qPCR results for microarray data verification.

Description: Comparison of gene expression measurements by microarray and qRT-PCR between *Z. mobilis* acetate-tolerant mutant AcR and wild-type ZM4 in the presence of 10 g/L NaAc in minimum media (MM) at different time points of 130, 148, 166 and 190 h post-inoculation. The numbers of X-axis and Y-axis are log_2_ based values of AcR versus ZM4 [log_2_(AcR/ZM4)] based on microarray and qPCR results respectively.

Additional file 8

File format: DOC

Title: Metabolic pathway differences between ZM4 and AcR.

Description: Metabolic pathway differences between ZM4 and AcR in MM with the supplementation of 10 g/L NaAc for genes with at least 2-fold significant difference between AcR and ZM4 (AcR/ZM4) (**A**), 120 and 107 proteins with at least 1.5-fold significant difference at 148 h (**B)** and 190 h (**C)** post-inoculation respectively, and an example of individual pathway with abundant enzymes identified in proteomic studies based on previous report [[1](#_ENREF_1)] and PathwayTools Omics Viewer result, the number after enzyme in the pathway is log2 transformed average spectral counts at 148 h post-inoculation (**D**).

Additional file 9

File format: DOC

Title: The information about the arrays used for comparison. Time: time point post-inoculation.

Description: The information for 44 microarrays from three experiments which were used for condition-specific gene identification.

Additional file 10

File format: DOC

Title: The numbers of significantly differentially expressed genes from 130 h to 190 h post-inoculation.

Description: The numbers of significantly differentially expressed genes in different time point comparison between AcR and ZM4 as well as the time course comparison for ZM4 or AcR from 130 h to 190 h post-inoculation.

Additional file 11

File format: EXCEL

Title: Transcriptomic profiling difference between ZM4 and AcR in different conditions.

Description: Transcriptomic profiling difference between *Z. mobilis* wild-type ZM4 and acetate-tolerant mutant AcR in different conditions. The ratio is log2 based value, bold font numbers are statistically significant and the ones highlighted with red color are upregulated and blue-highlighted ones are downregulated genes.

Additional file 12

File format: DOC

Title: The numbers of differentially expressed genes in different conditions.

Description: The numbers of significantly differentially expressed genes in different conditions of growth phase (exponential and stationary phase), Strain (AcR and ZM4), media (MM or RM), and stressors (NaCl and NaAc).

Additional file 13

File format: EXCEL

Title: Transcriptomic profiling difference between media and stressors.

Description: Transcriptomic profiling difference between MM and RM (**A**) and transcriptomic profiling differences with the presence of NaCl, NaAc and the differences between NaAc and NaCl (NaAc/NaCl) (**B**).

Additional file 14

File format: DOC

Title: The pre-documented protein interactions among upregulated and downregulated genes in MM.

Description: The interactions in STRING among 232 genes upregulated (**A**) and 247 genes downregulated (**B**) in MM compared to that of in RM.

Additional file 15

File format: DOC

Title: The pre-documented interaction among differentially expressed genes in response to different stressors.

Description: The pre-existing interaction among 47 genes upregulated (**A**) and 88 genes downregulated (**B**) in RM with NaCl treatment compared to that of in RM; 103 genes upregulated (**C**) and 159 genes downregulated (**D**) in RM with NaAc treatment compared to that of in RM; Venn diagrams of genes upregulated (**E**) or downregulated (**F**) in treatment comparison; 27 genes shared between those upregulated (**G**) and 65 genes shared between those down-regulated (**H**) in NaCl and in NaAc in RM; 37 genes upregulated (**I**) and 41 genes downregulated (**J**) in RM with NaAc treatment compared to that of NaCl treatment.

Additional file 16

File format: DOC

Title: The pre-documented interaction among differentially expressed genes in different growth phases.

Description: The pre-existing interaction among 68 upregulated (**A**) and 64 downregulated genes (**B**) in stationary phase compared to exponential phase in the presence of NaAc.

Additional file 17

File format: EXCEL

Title: The mean value of each genetic feature in 14 different conditions.

Description: The mean value of each genetic features in 14 different conditions for all *Z. mobilis* genetic features (**A**), data distributions and quartiles for 14 different conditions (**B**), and the mean value of each genetic features in 14 different conditions for the top 2.5% strongest ones (**C**),

Additional file 18

File format: DOC

Title: The hierarchical clustering result of all genetic features in 14 different conditions.

**Additional File 1. The effect of different concentration NaCl or NaAc on *Z. mobilis* growth in MM.**

**Additional File 3: Correlation between the proteomic difference of AcR versus ZM4 and the pre-documented interaction among common genes at time points of 148 h or 190 h post-inoculation.** Venn diagrams (**A-C**) of all the proteins identified (**A**); common upregulated ones (**B**); and common downregulated ones (**C**); interactions from STRINGS database (**D-E**) of 39 common upregulated (**D**) and 14 common down-regulated ones (**E**) at 148 and 190 h post-inoculation in AcR.


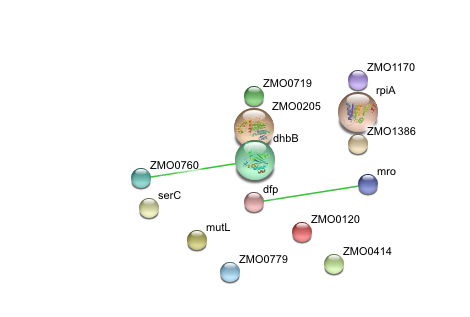

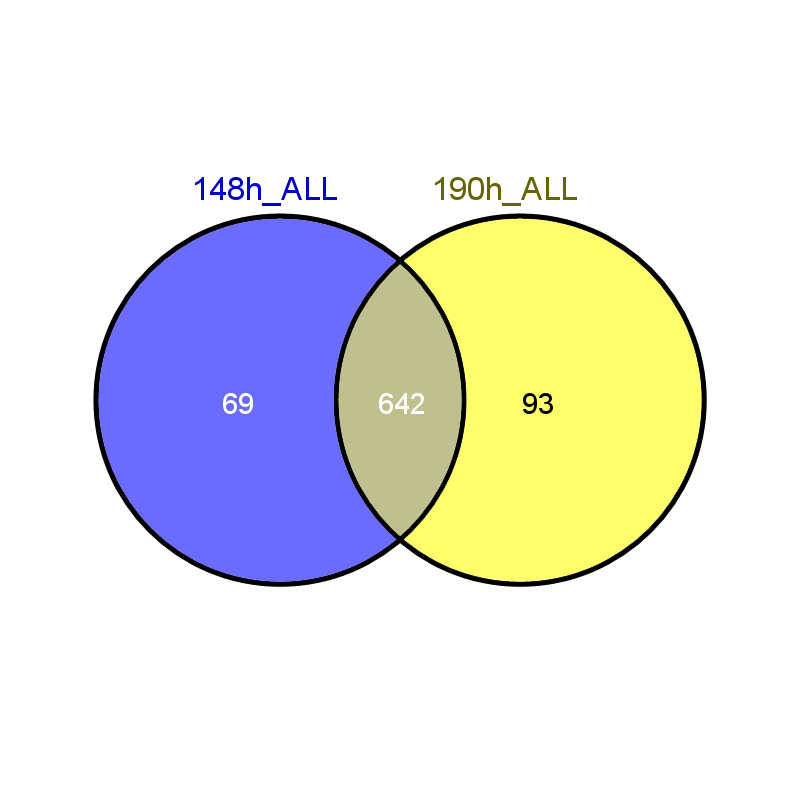

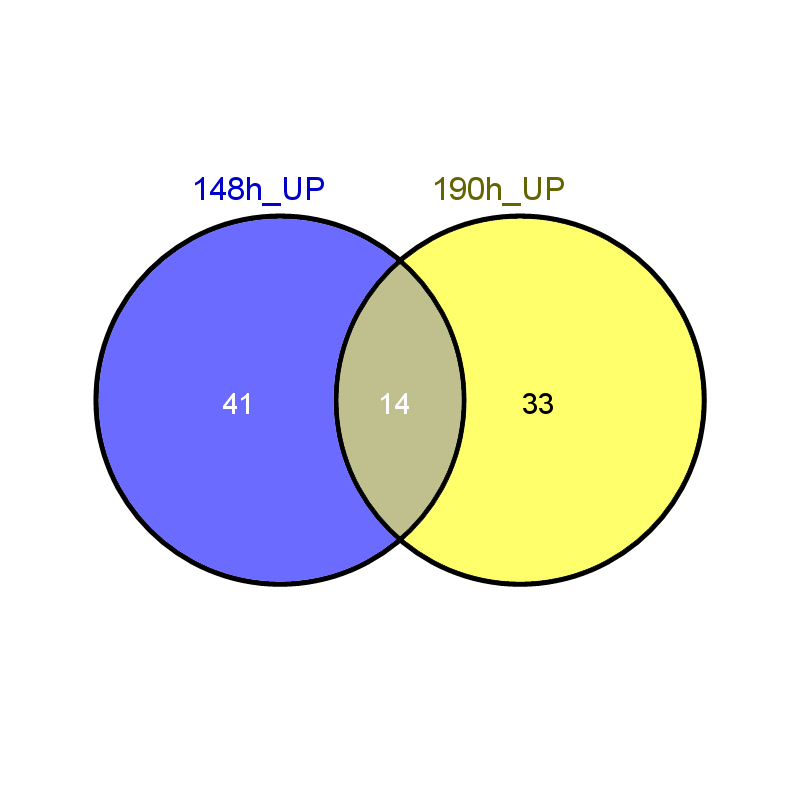

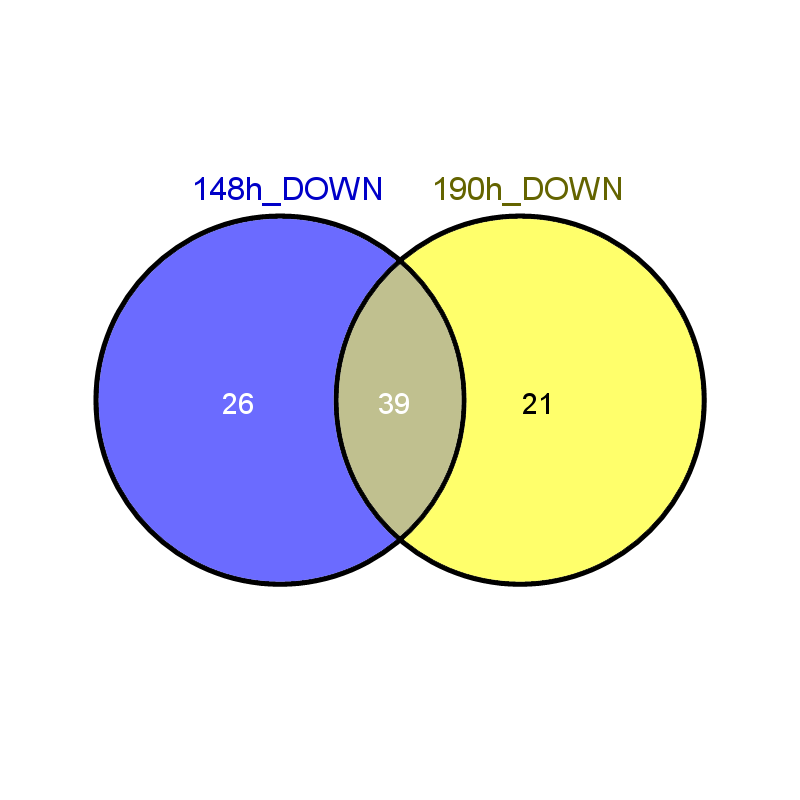


**A:**

**C:**

**B:**

**D:**


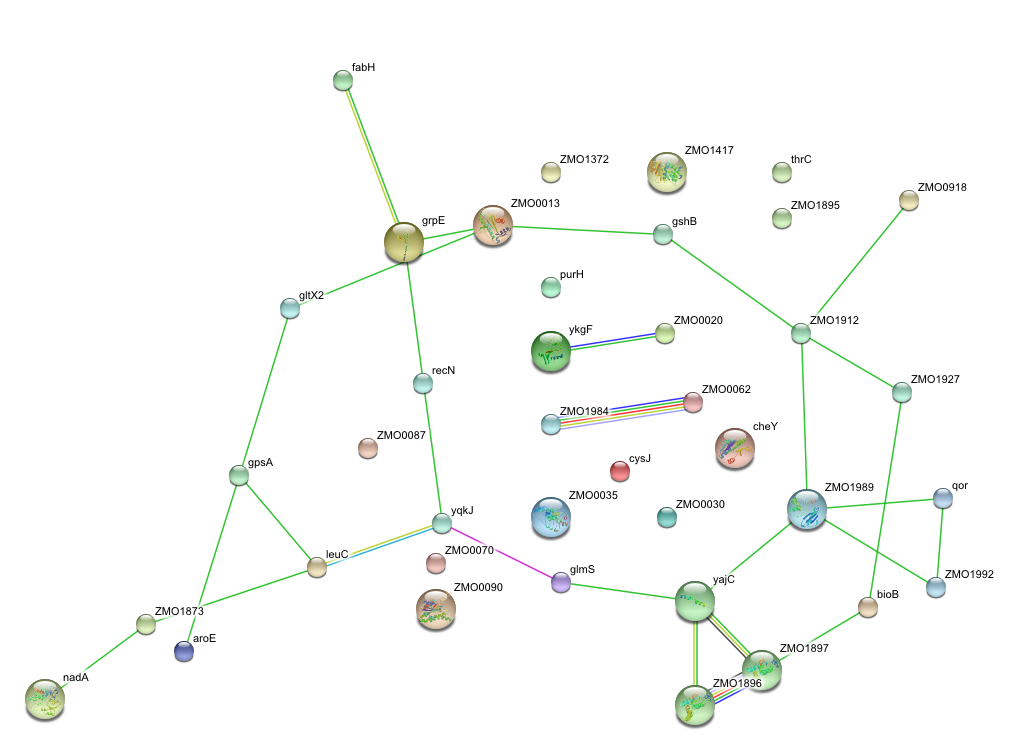


**E:**

**Additional File 4: Comparison the MW and pI distributions of 800 proteins (ca 46% of the total predicted proteins) identified from proteomics and the theoretical distribution based on genome prediction.**


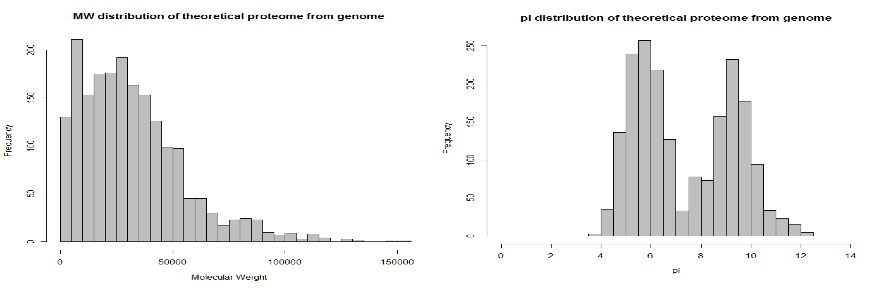


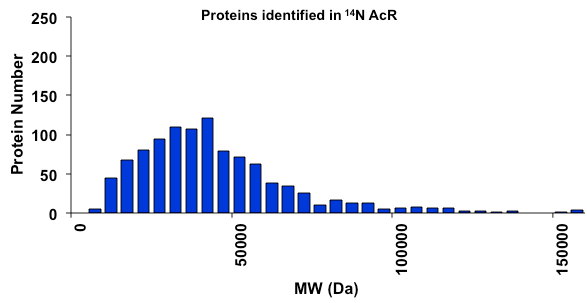

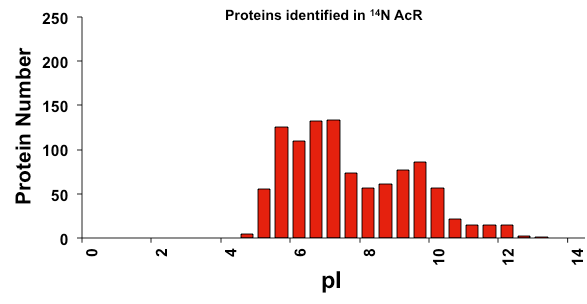


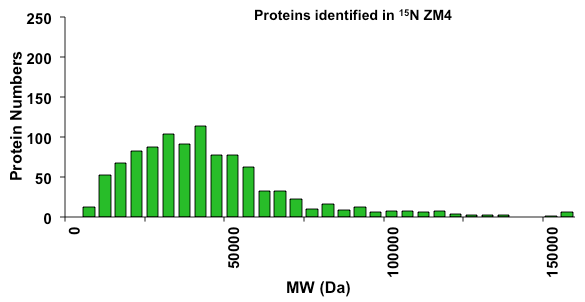

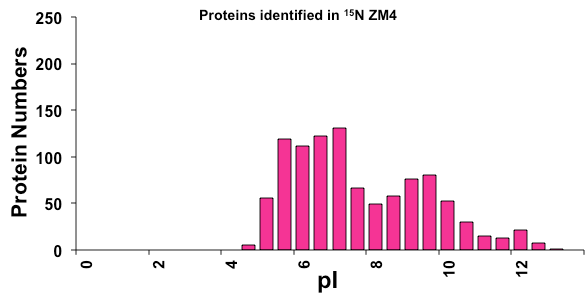


**Additional File 6. qPCR primers used in this study.**

| **PCR Primers** | **Gene_ID** | **Sequence (5' - 3')** | **Genome Position** |
| --- | --- | --- | --- |
| NT01ZM0113_qF | ZMO0119 | AACAAATTATTGCGCCTTGG |  |
| NT01ZM0113_qR |  | GGACATAGCAGACAGCGACA |  |
| NT01ZM0114_qF | ZMO0120 | CTTTTCATGCGGGTTTTGAT |  |
| NT01ZM0114_qR |  | CTTCGGGATGAATGGCTAAA |  |
| NT01ZM0336_qF | Intergenic Region | AAAAGCAAAGGCAACAGACG | 328069-328088 |
| NT01ZM0336_qR |  | ACGGTAGCAGGGAAGGTTCT | 327999-328018 |
| NT01ZM0379_qF | ZMO0374 | AAGAGGAAATTGGCCCTGTT |  |
| NT01ZM0379_qR |  | TTAAGGGCCTGTGCAATACC |  |
| NT01ZM0387_qF | ZMO0381 | AATCGGCACCCGTTATATCC |  |
| NT01ZM0387_qR |  | TAGCCATCGCCAAACGTAAT |  |
| NT01ZM0401_qF | ZMO0395 | TGTCACCACTATTCGCCAAA |  |
| NT01ZM0401_qR |  | TGCGGTCTGCTATCAATCAG |  |
| NT01ZM0647_qF | ZMO0631 | CGGAAATCCTGATGGAAAAA |  |
| NT01ZM0647_qR |  | CATTTGTTCAGCCACAATGG |  |
| NT01ZM0961_qF | ZMO0930 | TCAGCAAGAACAAGGCGATA |  |
| NT01ZM0961_qR |  | ATCAGCATTGCCACAGACAG |  |
| NT01ZM1634_qF | ZMO1572 | TTATTGGGTGCTGGATGGTT |  |
| NT01ZM1634_qR |  | CAGACGAGCAAAAGGCTACC |  |

**Additional File 7:** The correlation between microarray and qPCR results for microarray data verification. Comparison of gene expression measurements by microarray and qRT-PCR between *Z. mobilis* acetate-tolerant mutant AcR and wild-type ZM4 in the presence of 10 g/L NaAc in minimum media (MM) at different time points of 130, 148, 166 and 190 h post-inoculation. The numbers of X-axis and Y-axis are log_2_ based values of AcR versus ZM4 [log_2_(AcR/ZM4)] based on microarray and qPCR results respectively.

**Additional File 8.** Metabolic pathway difference between ZM4 and AcR in MM with the supplementation of 10 g/L NaAc for genes with at least 2-fold significant difference between AcR and ZM4 (AcR/ZM4) (**A**), 120 and 107 proteins with at least 1.5-fold significant difference at 148 h (**B)** and 190 h post-inoculation respectively (**C)**, and an example of individual pathway with abundant enzymes identified in proteomic studies based on previous report [[1](#_ENREF_1)] and PathwayTools Omics Viewer result, the number after enzyme in the pathway is log2 transformed average spectral counts at 148 h and 190 h post-inoculation (**D**). The red boxes show examples of pathway difference with different comparisons.


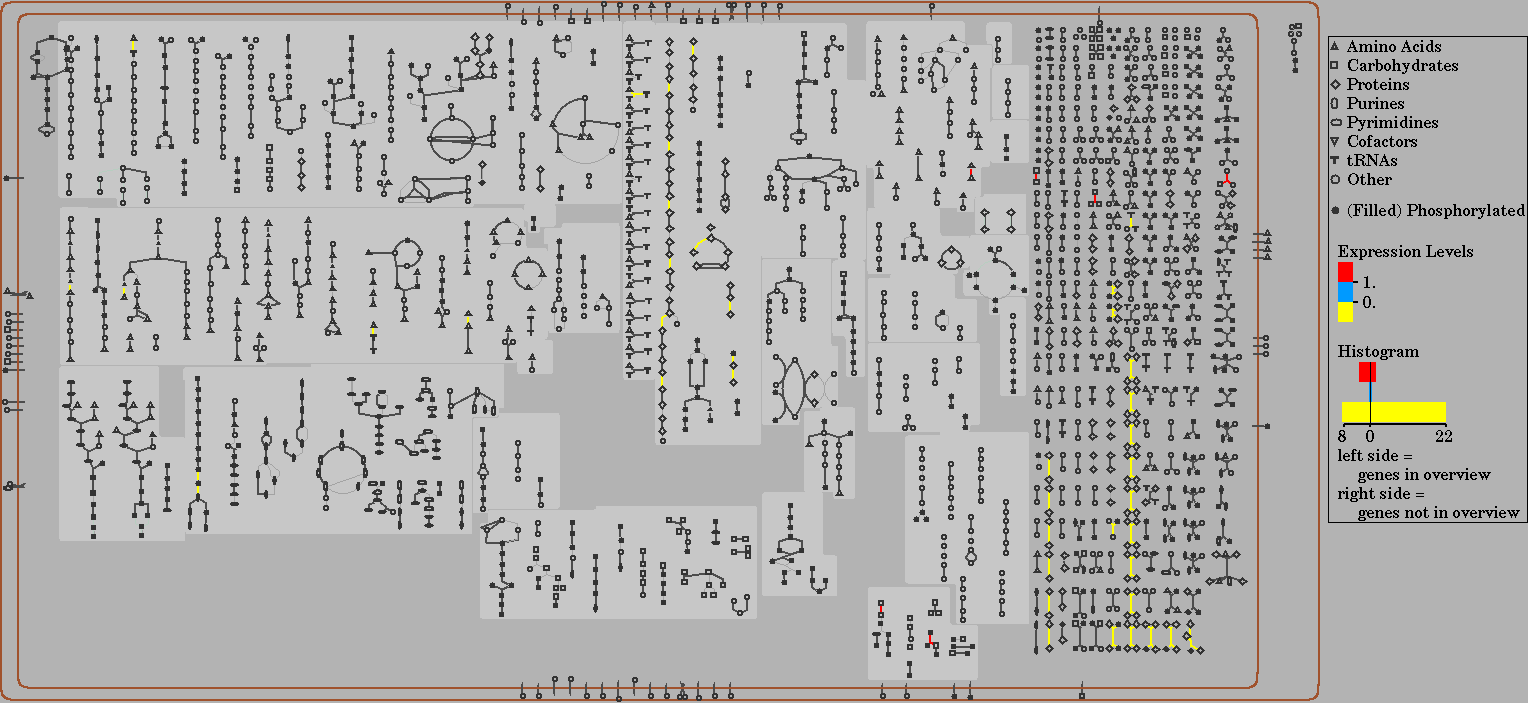


**Hopanoid synthesis**

**Glycolysis**

**Mixed Acid Production**

**A**


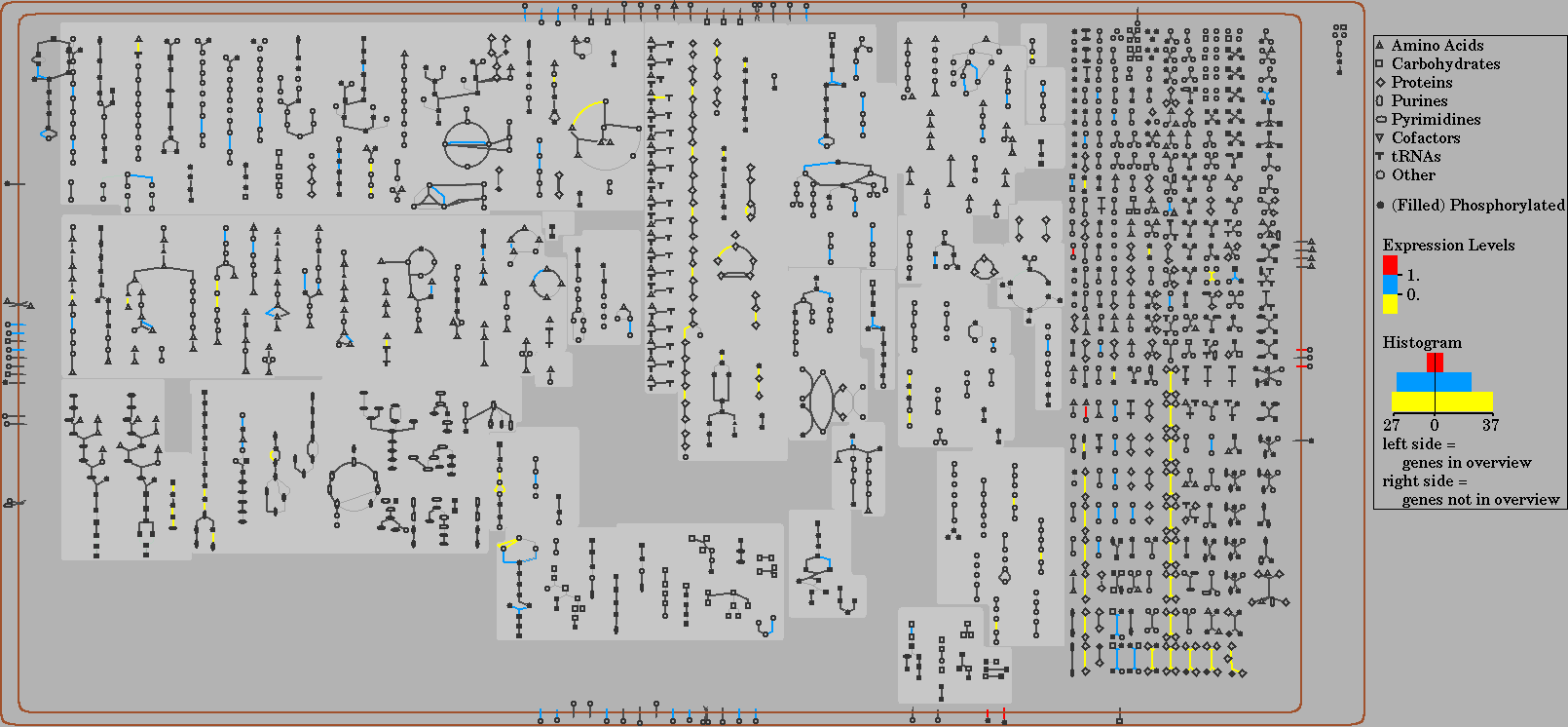


**Hopanoid synthesis**

**Glycolysis**

**Mixed Acid Production**

**B**


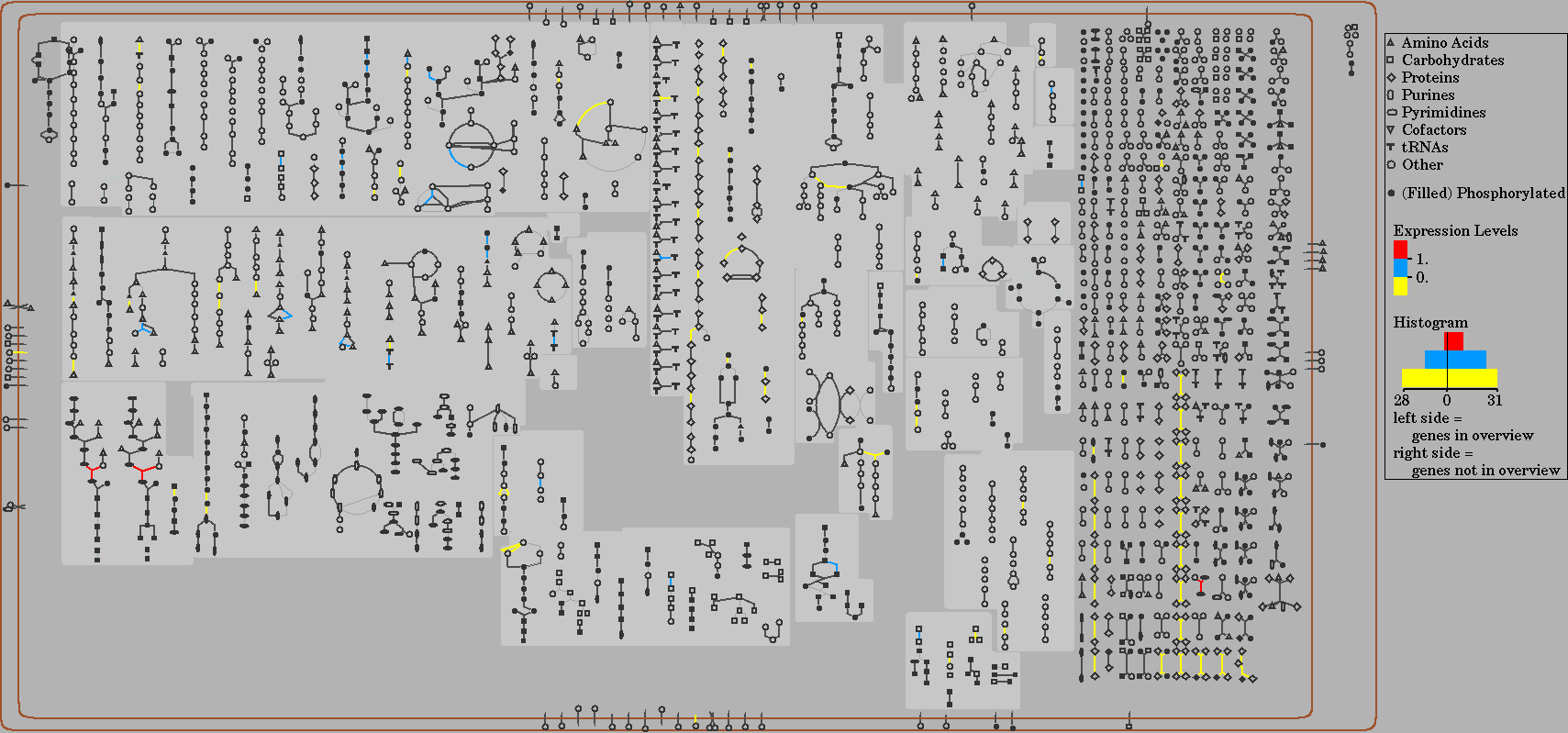


**Glycolysis**

**Mixed Acid Production**

**Hopanoid synthesis**

**Mixed Acid Production**

**Hopanoid synthesis**

**Mixed Acid Production**

**Glycolysis**

**Hopanoid synthesis**

**C**


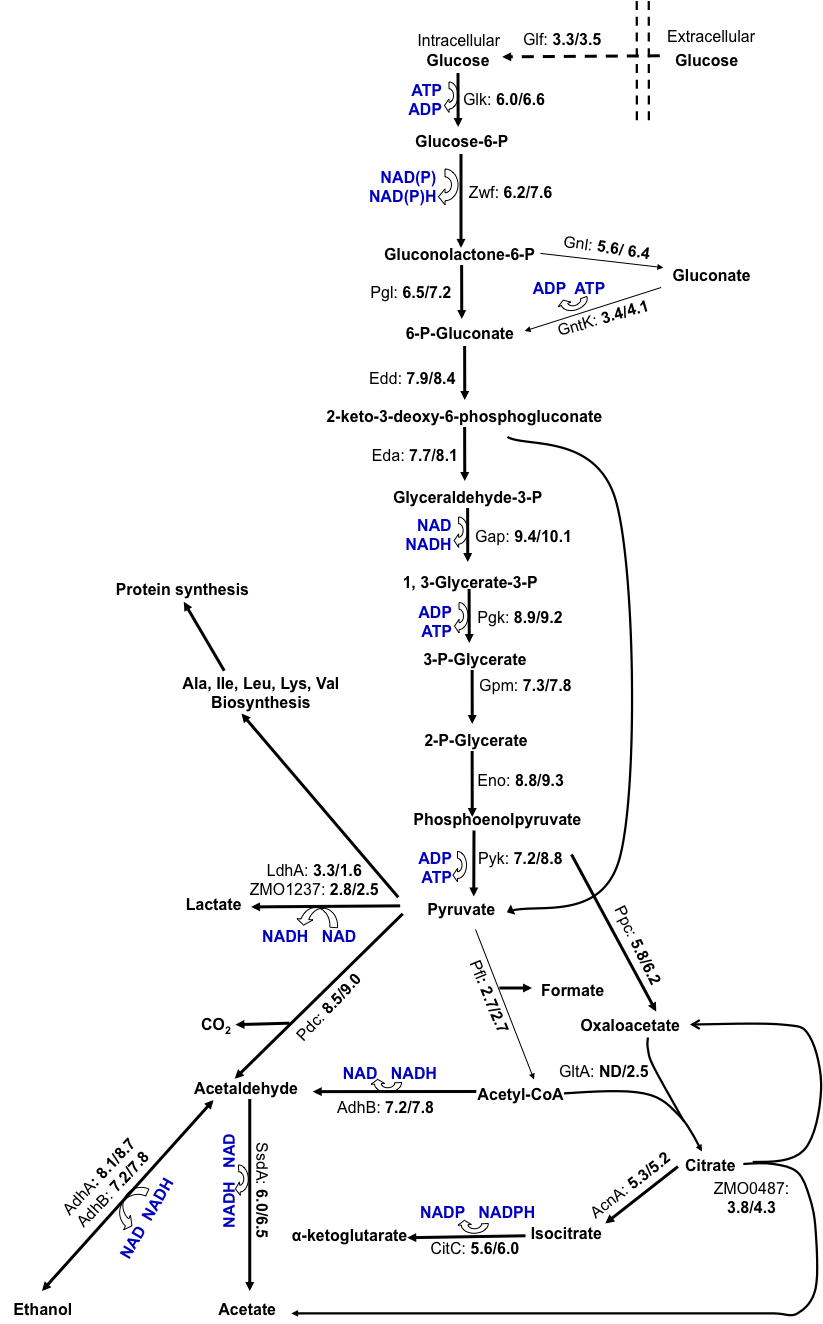


**D**

**Additional File 9.** The information about the arrays used for comparison.

| **CHIP_ID** | **DYE** | **Fermentor** | **Strain** | **Media** | **Treatment** | **Time** | **Phase** | **File** |
| --- | --- | --- | --- | --- | --- | --- | --- | --- |
| **ZM4 Time Course Study in RM** | | | | | | | | |
| 10259402 | Cy3 | F4 | ZM4 | RM | Control | 6h | Exponential | 10259402_532_calls.txt |
| 10259702 | Cy3 | F1 | ZM4 | RM | Control | 6h | Exponential | 10259702_532_calls.txt |
| 10465402 | Cy3 | F4 | ZM4 | RM | Control | 10h | Stationary | 10465402_532_calls.txt |
| 10465502 | Cy3 | F1 | ZM4 | RM | Control | 10h | Stationary | 10465502_532_calls.txt |
| 10466702 | Cy3 | F4 | ZM4 | RM | Control | 13.5h | Stationary | 10466702_532_calls.txt |
| 10467002 | Cy3 | F1 | ZM4 | RM | Control | 13.5h | Stationary | 10467002_532_calls.txt |
| 10494102 | Cy3 | F4 | ZM4 | RM | Control | 26h | Stationary | 10494102_532_calls.txt |
| 10494402 | Cy3 | F1 | ZM4 | RM | Control | 26h | Stationary | 10494402_532_calls.txt |
| **ZM4 and AcR Time Course Study in MM with NaAc supplementation (Bolded samples used for proteomics)** | | | | | | | | |
| 8523302 | Cy3 | F5 | AcR | MM | NaAc 10g/L | 130h | Exponential | 8523302_532_calls.txt |
| 8523202 | Cy3 | F6 | AcR | MM | NaAc 10g/L | 130h | Exponential | 8523202_532_calls.txt |
| 8523502 | Cy3 | F2 | ZM4 | MM | NaAc 10g/L | 130h | Exponential | 8523502_532_calls.txt |
| 8523402 | Cy3 | F3 | ZM4 | MM | NaAc 10g/L | 130h | Exponential | 8523402_532_calls.txt |
| **8524702** | **Cy3** | **F5** | **AcR** | **MM** | **NaAc 10g/L** | **148h** | **Stationary** | **8524702_532_calls.txt** |
| **8524602** | **Cy3** | **F6** | **AcR** | **MM** | **NaAc 10g/L** | **148h** | **Stationary** | **8524602_532_calls.txt** |
| **8524902** | **Cy3** | **F2** | **ZM4** | **MM** | **NaAc 10g/L** | **148h** | **Exponential** | **8524902_532_calls.txt** |
| **8891802** | **Cy3** | **F3** | **ZM4** | **MM** | **NaAc 10g/L** | **148h** | **Exponential** | **8891802_532_calls.txt** |
| 8529902 | Cy3 | F5 | AcR | MM | NaAc 10g/L | 166h | Stationary | 8529902_532_calls.txt |
| 8529802 | Cy3 | F6 | AcR | MM | NaAc 10g/L | 166h | Stationary | 8529802_532_calls.txt |
| 8530102 | Cy3 | F2 | ZM4 | MM | NaAc 10g/L | 166h | Stationary | 8530102_532_calls.txt |
| 8891702 | Cy3 | F3 | ZM4 | MM | NaAc 10g/L | 166h | Stationary | 8891702_532_calls.txt |
| **8536802** | **Cy3** | **F5** | **AcR** | **MM** | **NaAc 10g/L** | **190h** | **Stationary** | **8536802_532_calls.txt** |
| **8536702** | **Cy3** | **F6** | **AcR** | **MM** | **NaAc 10g/L** | **190h** | **Stationary** | **8536702_532_calls.txt** |
| **8537002** | **Cy3** | **F2** | **ZM4** | **MM** | **NaAc 10g/L** | **190h** | **Stationary** | **8537002_532_calls.txt** |
| **8536902** | **Cy3** | **F3** | **ZM4** | **MM** | **NaAc 10g/L** | **190h** | **Stationary** | **8536902_532_calls.txt** |
| **ZM4 and AcR Time Course Study in RM with NaAc supplementation** | | | | | | | | |
| 2164802 | Cy3 | F5 | AcR | RM | NaAc 12g/L | 4h | Exponential | 2164802_532_calls.txt |
| 2164402 | Cy3 | F6 | AcR | RM | NaAc 12g/L | 4h | Exponential | 2164402_532_calls.txt |
| 2163702 | Cy3 | F1 | ZM4 | RM | NaAc 12g/L | 4h | Exponential | 2163702_532_calls.txt |
| 2164302 | Cy3 | F3 | ZM4 | RM | NaAc 12g/L | 4h | Exponential | 2164302_532_calls.txt |
| 2165202 | Cy3 | F5 | AcR | RM | NaAc 12g/L | 6.5h | Exponential | 2165202_532_calls.txt |
| 2165302 | Cy3 | F6 | AcR | RM | NaAc 12g/L | 6.5h | Exponential | 2165302_532_calls.txt |
| 2164702 | Cy3 | F1 | ZM4 | RM | NaAc 12g/L | 6.5h | Exponential | 2164702_532_calls.txt |
| 2164902 | Cy3 | F3 | ZM4 | RM | NaAc 12g/L | 6.5h | Exponential | 2164902_532_calls.txt |
| 2165702 | Cy3 | F5 | AcR | RM | NaAc 12g/L | 24h | Stationary | 2165702_532_calls.txt |
| 2165802 | Cy3 | F6 | AcR | RM | NaAc 12g/L | 24h | Stationary | 2165802_532_calls.txt |
| 2165402 | Cy3 | F1 | ZM4 | RM | NaAc 12g/L | 24h | Stationary | 2165402_532_calls.txt |
| 2165502 | Cy3 | F3 | ZM4 | RM | NaAc 12g/L | 24h | Stationary | 2165502_532_calls.txt |
| **ZM4 and AcR Time Course Study in RM with NaCl supplementation** | | | | | | | | |
| 2166302 | Cy3 | F2 | AcR | RM | NaCl 8.6g/L | 5h | Exponential | 2166302_532_calls.txt |
| 2166502 | Cy3 | F4 | AcR | RM | NaCl 8.6g/L | 5h | Exponential | 2166502_532_calls.txt |
| 2166002 | Cy3 | F1 | ZM4 | RM | NaCl 8.6g/L | 5h | Exponential | 2166002_532_calls.txt |
| 2166102 | Cy3 | F3 | ZM4 | RM | NaCl 8.6g/L | 5h | Exponential | 2166102_532_calls.txt |
| 2167202 | Cy3 | F2 | AcR | RM | NaCl 8.6g/L | 24h | Stationary | 2167202_532_calls.txt |
| 2167302 | Cy3 | F4 | AcR | RM | NaCl 8.6g/L | 24h | Stationary | 2167302_532_calls.txt |
| 2166802 | Cy3 | F1 | ZM4 | RM | NaCl 8.6g/L | 24h | Stationary | 2166802_532_calls.txt |
| 2167002 | Cy3 | F3 | ZM4 | RM | NaCl 8.6g/L | 24h | Stationary | 2167002_532_calls.txt |

**Additional File 10. The numbers of significantly differentially expressed genes in different time point comparison between AcR and ZM4 as well as the time course comparison for ZM4 or AcR from 130 h to 190 h post-inoculation.** AcR/ZM4: gene expression difference between acetate-tolerant mutant AcR and wild-type ZM4 at different time points of 130, 148, 166, and 190 h. Up: number of upregulated genes; Down: number of downregulated genes. AcR_TC and ZM4_TC: time course comparison of gene expression within AcR or ZM4 background from time point of 130 h to 190 h post-inoculation respectively.

| **1. Difference between AcR and at different time points** | | | |
| --- | --- | --- | --- |
|  |  | **AcR/ZM4** |  |
| **130 h** | Up | 39 |  |
|  | Down | 43 |  |
| **148 h** | Up | 78 |  |
|  | Down | 150 |  |
| **166 h** | Up | 7 |  |
|  | Down | 36 |  |
| **190 h** | Up | 2 |  |
|  | Down | 28 |  |
| **2. Time course comparison** | |  |  |
|  |  | **AcR_TC** | **ZM4_TC** |
| **130-148 h** | Up | 136 | 13 |
|  | Down | 73 | 9 |
| **148-166 h** | Up | 0 | 46 |
|  | Down | 0 | 13 |
| **166-190 h** | Up | 0 | 0 |
|  | Down | 0 | 0 |
| **130-190 h** | Up | 145 | 149 |
|  | Down | 82 | 72 |

**Additional File 12.** **The numbers of differentially expressed genes in different conditions. MM**: minimum media; **RM**: rich media; **NaAc**: sodium acetate; **NaCl**: sodium chloride; **ZM4**: *Z. mobilis* wild-type ZM4; **AcR**: acetate tolerant mutant derived from ZM4; **RM NaCl**: RM with the supplementation of NaCl; **RM NaAc**: RM with the supplementation of NaAc; **MM NaAc**: MM with the supplementation of NaAc; **RM All**: combine the condition of RM, RM NaCl, and RM NaAc. **Log**: exponential phase comparison; **Stat**: stationary phase comparison; **All**: comparison combining both exponential and Stationary phases. **Stat/Log**: stationary phase versus exponential phase for phase comparison; **AcR/ZM4**: AcR versus ZM4 for strain comparison; **MM/RM**: MM versus RM for media comparison; **NaAc/NaCl**, **NaCl/RM**, and **NaAc/RM**: NaAc versus NaCl, NaCl versus RM and NaAc versus RM respectively for treatment comparisons. **Up**: number of significantly upregulated genes with at least 2-fold change; **Down**: number of significantly downregulated genes with at least 2-fold change; **Total**: the sum of Up and Down.

| **1. Phase:** compare growth phase difference of AcR and ZM4 in the presence of NaAc or NaCl in RM or MM | | | | | | | | | | | | | | | | | | | | | | | | | |
| --- | --- | --- | --- | --- | --- | --- | --- | --- | --- | --- | --- | --- | --- | --- | --- | --- | --- | --- | --- | --- | --- | --- | --- | --- | --- |
| **Stat/Log** | **NaAc** | | **NaAc (MM)** | | **NaAc (RM)** | | **NaCl (RM)** | | **ZM4 (RM All)** | | **ZM4 (RM Only)** | | **ZM4 (RM NaCl)** | | **ZM4 (RM NaAc)** | | **ZM4 (MM NaAc)** | | **ZM4 (NaAc)** | | **AcR (RM NaCl)** | | **AcR (RM NaAc)** | **AcR (MM NaAc)** | **AcR (NaAc)** |
| **Up** | 68 | | 62 | | 184 | | 315 | | 224 | | 291 | | 313 | | 135 | | 56 | | 68 | | 332 | | 243 | 114 | 100 |
| **Down** | 64 | | 91 | | 147 | | 346 | | 229 | | 395 | | 347 | | 93 | | 88 | | 41 | | 358 | | 219 | 124 | 78 |
| **Total** | 132 | | 153 | | 331 | | 661 | | 453 | | 686 | | 660 | | 228 | | 144 | | 109 | | 690 | | 462 | 238 | 178 |
| **2. Strain:** compare difference between AcR and ZM4 in different conditions | | | | | | | | | | | | | | | | | | | | | | | |  |  |
|  | | **NaAc** | | | | | | **NaAc MM** | | | | | | **NaAc RM** | | | | | | **NaCl RM** | | | |  |  |
| **AcR/ZM4** | | **All** | | **Log** | | **Stat** | | **All** | | **Log** | | **Stat** | | **All** | | **Log** | | **Stat** | | **All** | | **Log** | **Stat** |  |  |
| **Up** | | 2 | | 24 | | 5 | | 6 | | 25 | | 3 | | 8 | | 40 | | 29 | | 4 | | 3 | 43 |  |  |
| **Down** | | 3 | | 8 | | 4 | | 18 | | 22 | | 32 | | 11 | | 34 | | 14 | | 15 | | 18 | 42 |  |  |
| **Total** | | 5 | | 32 | | 9 | | 24 | | 47 | | 35 | | 19 | | 74 | | 43 | | 19 | | 21 | 85 |  |  |
| **3. Media:** compare difference between RM and MM in the presence of NaAc for AcR and ZM4 | | | | | | | | | | | | | | | | | | | | | |  |  |  |  |
|  | | **NaAc** | | | | | | **AcR NaAc** | | | | | | **ZM4 NaAc** | | | | | |  |  |  |  |  |  |
| **MM/RM** | | **All** | | **Log** | | **Stat** | | **All** | | **Log** | | **Stat** | | **All** | | **Log** | | **Stat** | |  |  |  |  |  |  |
| **Up** | | 232 | | 270 | | 304 | | 250 | | 289 | | 331 | | 246 | | 260 | | 282 | |  |  |  |  |  |  |
| **Down** | | 247 | | 281 | | 294 | | 260 | | 295 | | 361 | | 250 | | 277 | | 276 | |  |  |  |  |  |  |
| **Total** | | 479 | | 551 | | 598 | | 510 | | 584 | | 692 | | 496 | | 537 | | 558 | |  |  |  |  |  |  |
| **4-1. Treatment:** compare difference between NaAc and NaCl for AcR and ZM4 in RM | | | | | | | | | | | | | | | | | | | |  |  |  |  |  |  |
|  | | **RM** | | | | | | **ZM4 RM** | | | | | | **AcR RM** | | | | | |  |  |  |  |  |  |
| **NaAc/NaCl** | | **All** | | **Log** | | **Stat** | | **All** | | **Log** | | **Stat** | | **All** | | **Log** | | **Stat** | |  |  |  |  |  |  |
| **Up** | | 37 | | 25 | | 130 | | 41 | | 39 | | 147 | | 52 | | 34 | | 151 | |  |  |  |  |  |  |
| **Down** | | 41 | | 58 | | 131 | | 43 | | 64 | | 144 | | 52 | | 60 | | 150 | |  |  |  |  |  |  |
| **Total** | | 78 | | 83 | | 261 | | 84 | | 103 | | 291 | | 104 | | 94 | | 301 | |  |  |  |  |  |  |
| **4-2. Treatment:** compare effect of NaAc or NaCl on ZM4 in RM | | | | | | | | | | | | | | | | | | | |  |  |  |  |  |  |
|  | | **NaCl/RM** | | | | | | **NaAc/RM** | | | | | |  |  |  |  |  |  |  |  |  |  |  |  |
| **ZM4** | | **All** | | **Log** | | **Stat** | | **All** | | **Log** | | **Stat** | |  |  |  |  |  |  |  |  |  |  |  |  |
| **Up** | | 47 | | 41 | | 220 | | 103 | | 141 | | 212 | |  |  |  |  |  |  |  |  |  |  |  |  |
| **Down** | | 88 | | 62 | | 190 | | 159 | | 193 | | 218 | |  |  |  |  |  |  |  |  |  |  |  |  |
| **Total** | | 135 | | 103 | | 410 | | 262 | | 334 | | 430 | |  |  |  |  |  |  |  |  |  |  |  |  |

**Additional File 14.** The interactions in STRING among 232 genes upregulated (**A**) and 247 genes downregulated (**B**) in MM compared to that of in RM.


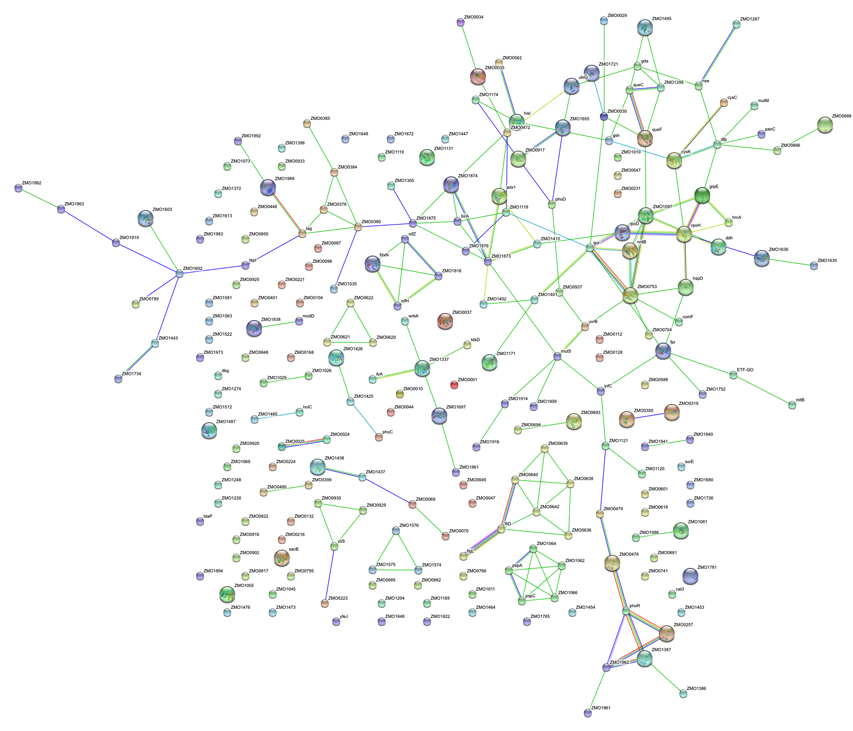


**A:**


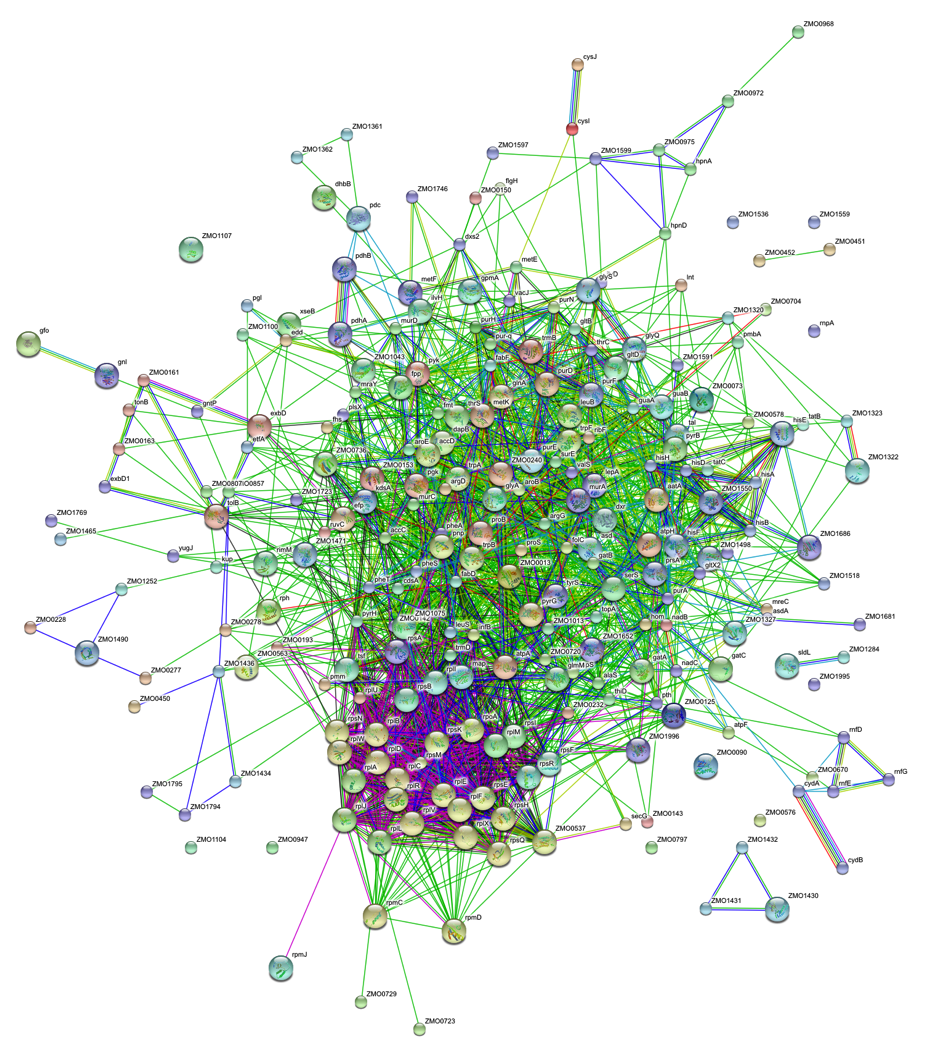


**B:**

**Additional File 15.** The pre-existing interaction among 47 genes upregulated (**A**) and 88 genes downregulated (**B**) in RM with NaCl treatment compared to that of in RM; 103 genes upregulated (**C**) and 159 genes downregulated (**D**) in RM with NaAc treatment compared to that of in RM; Venn diagrams of genes upregulated (**E**) or downregulated (**F**) in treatment comparison; 27 genes shared between those upregulated (**G**) and 65 genes shared between those down-regulated (**H**) in NaCl and in NaAc in RM; 37 genes upregulated (**I**) and 41 genes downregulated (**J**) in RM with NaAc treatment compared to that of NaCl treatment.


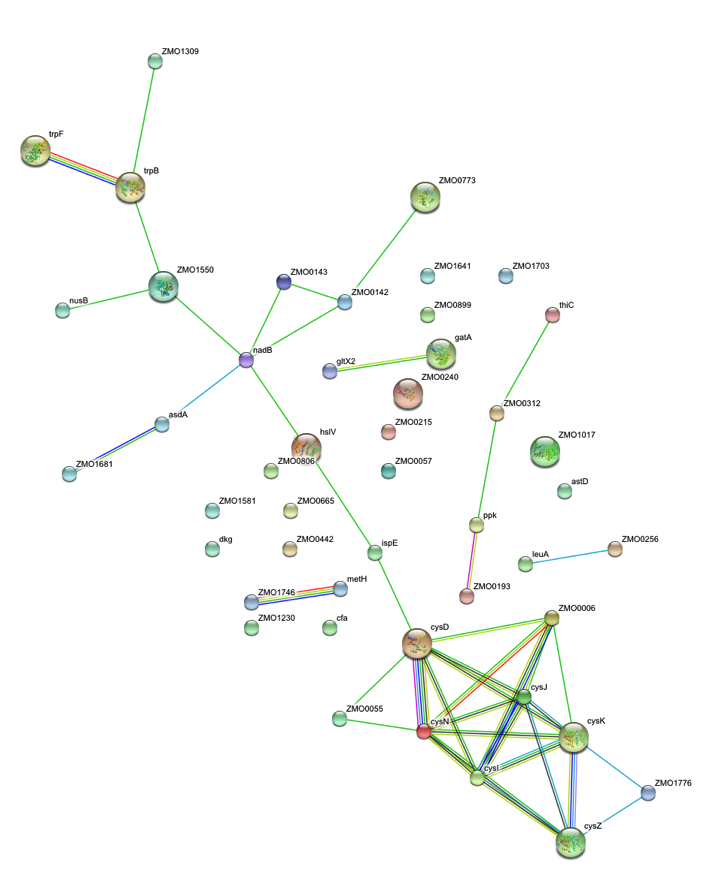

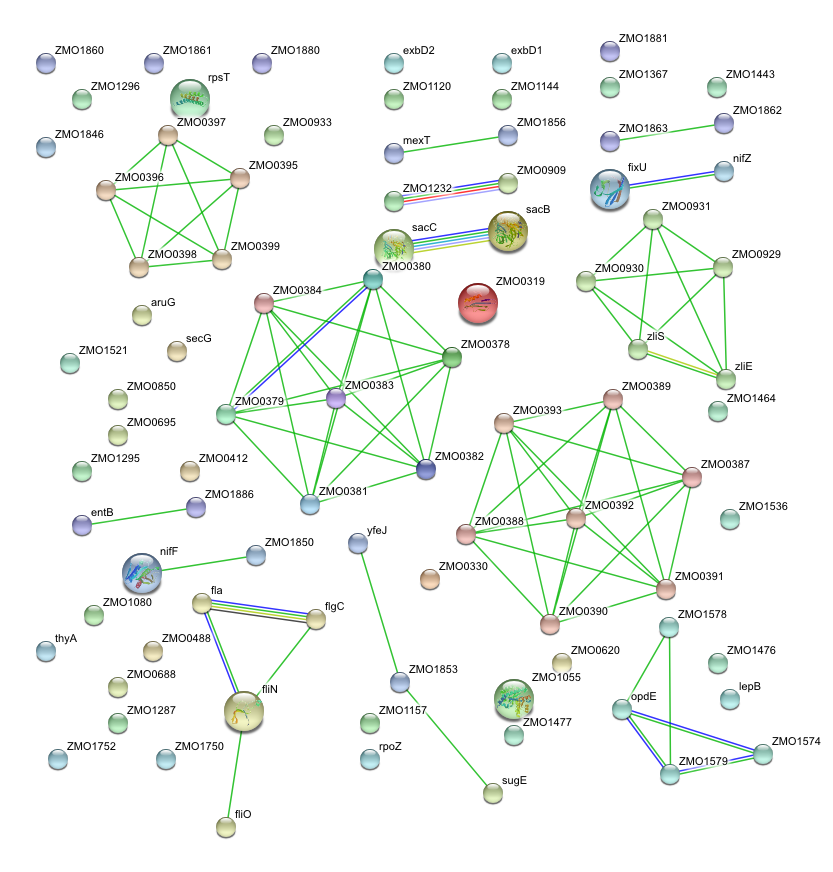


**A:**

**B:**


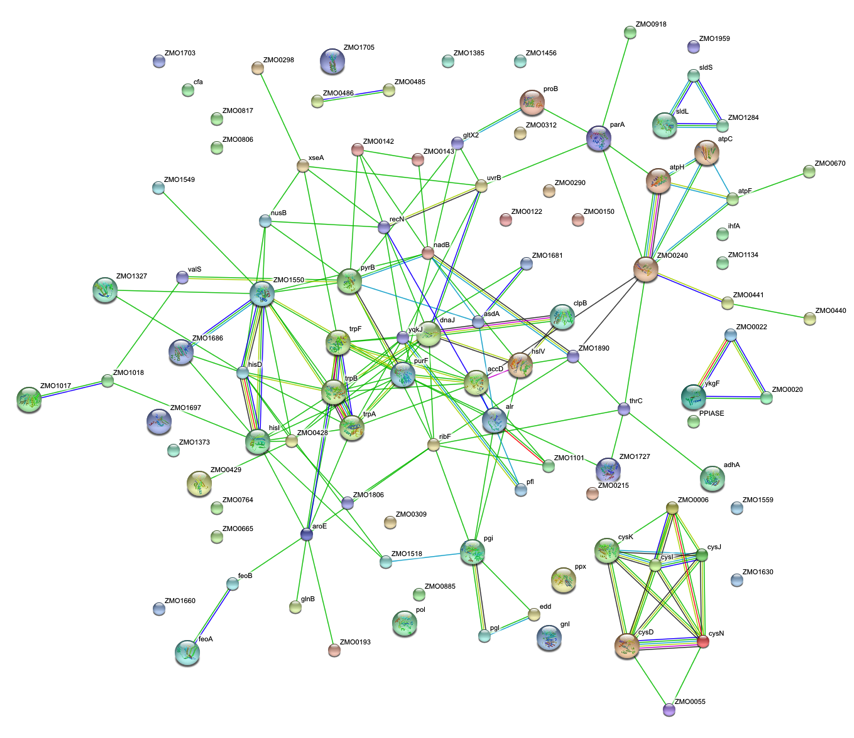

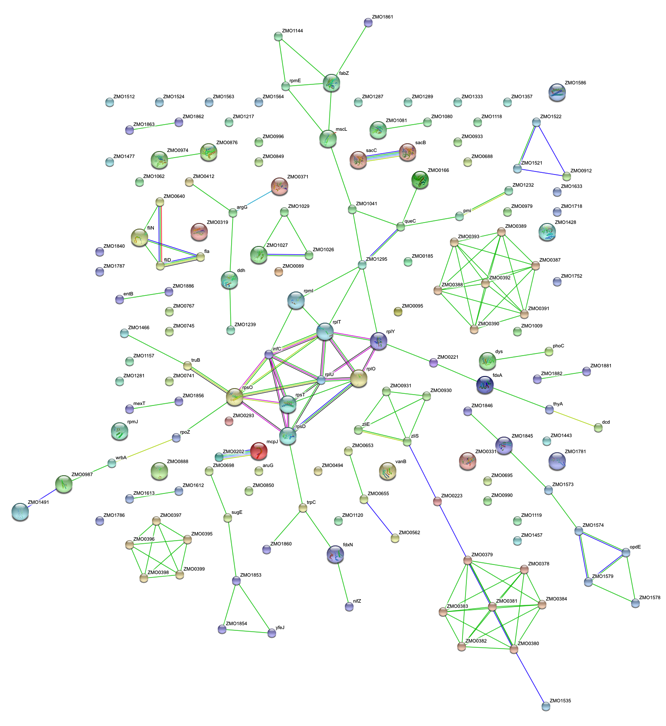


**C:**

**D:**


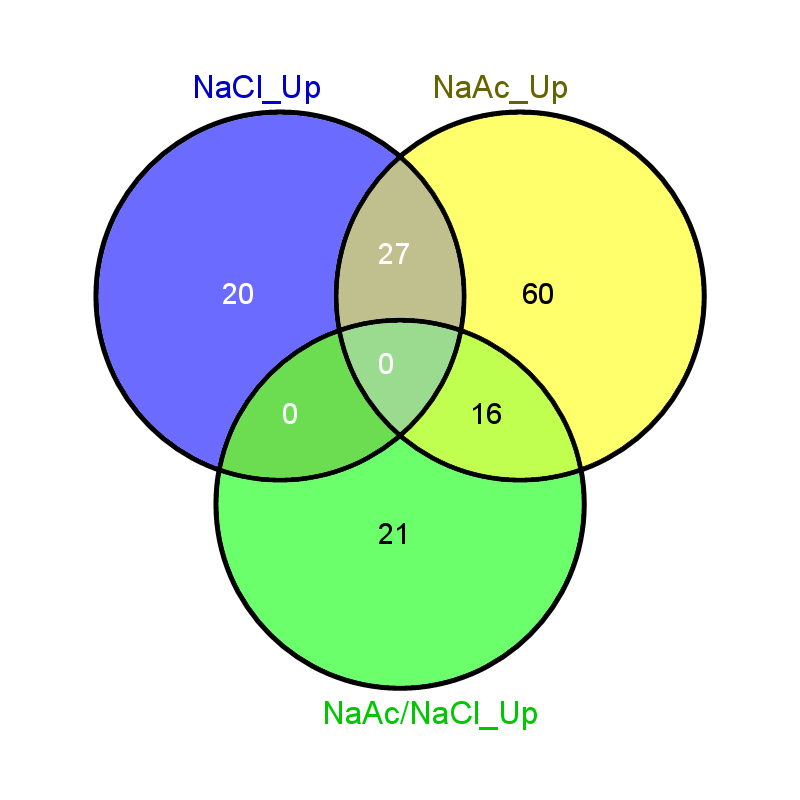

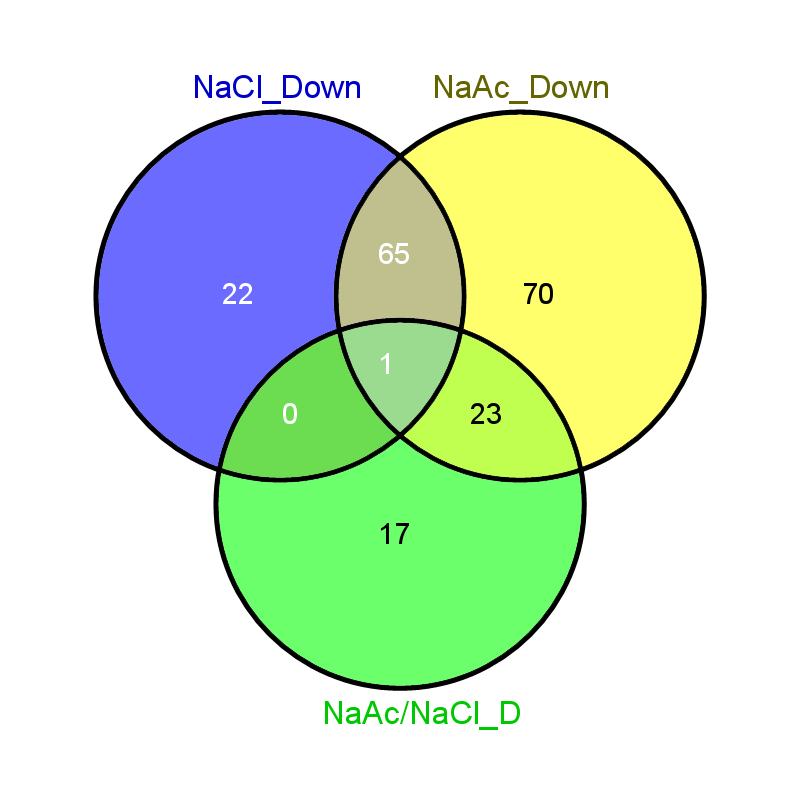


**F:**

**E:**


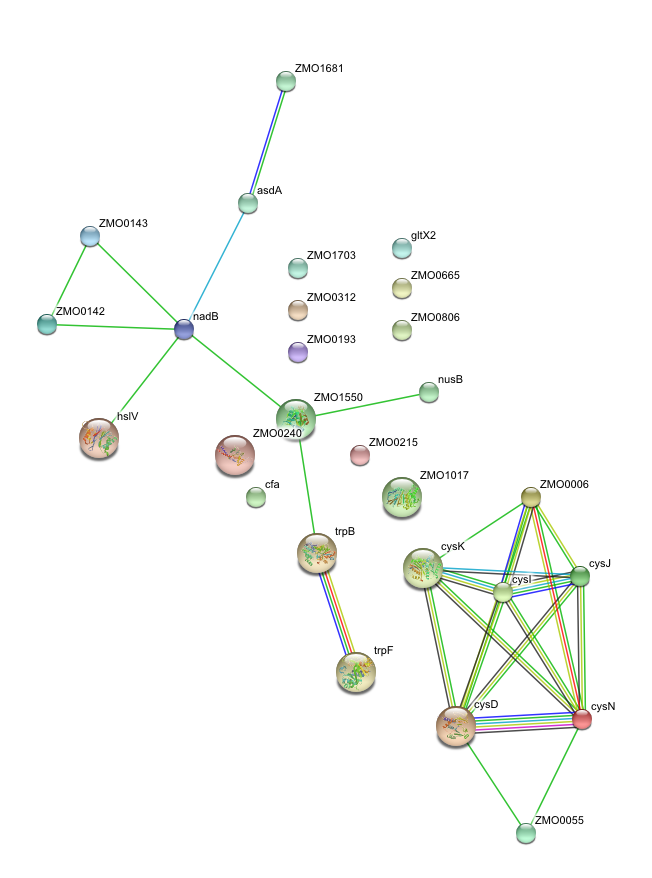

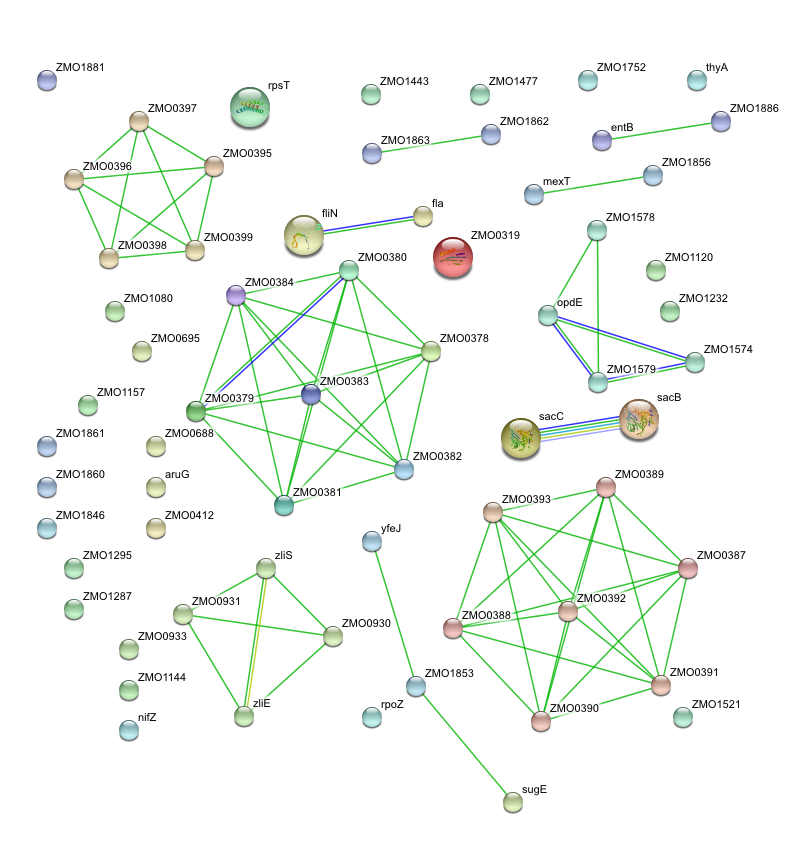

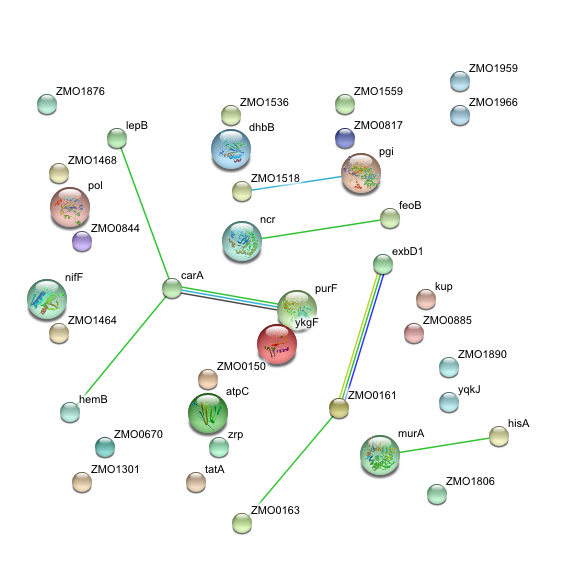

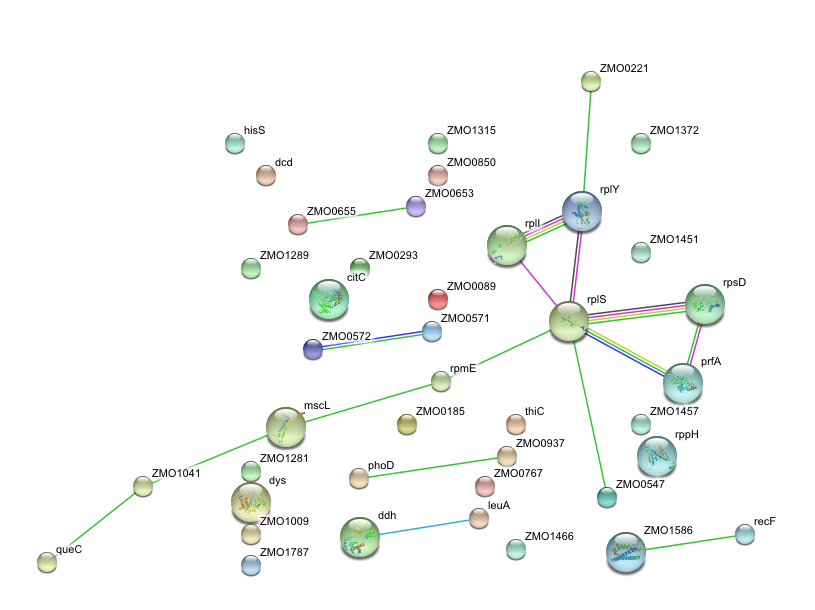
**Additional File 16.** The pre-existing interaction among 68 upregulated (**A**) and 64 downregulated genes (**B**) in stationary phase compared to exponential phase in the presence of NaAc.

**J:**

**I:**

**H:**

**G:**


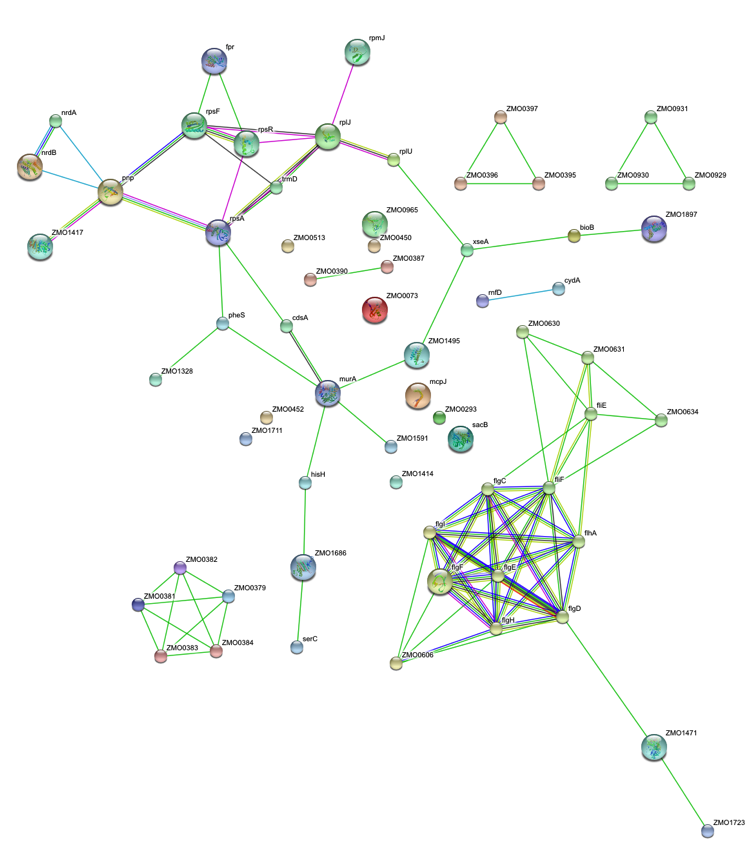

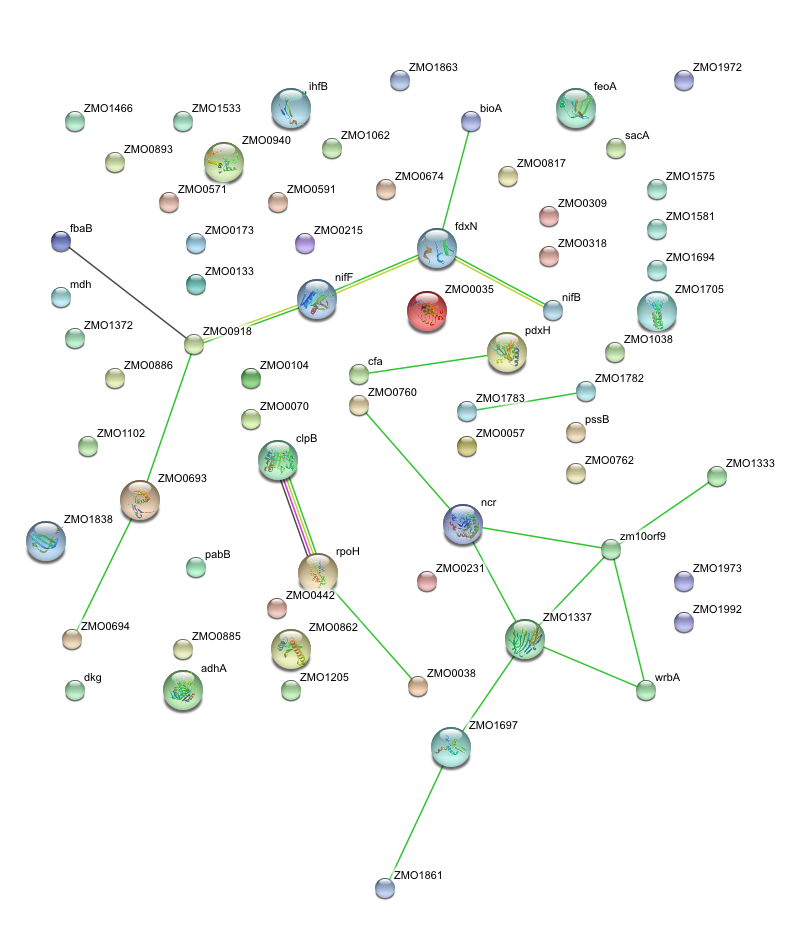


**B:**

**A:**

Additional file 18

File format: DOC

Title: The hierarchical clustering result of all genetic features in 14 different conditions.


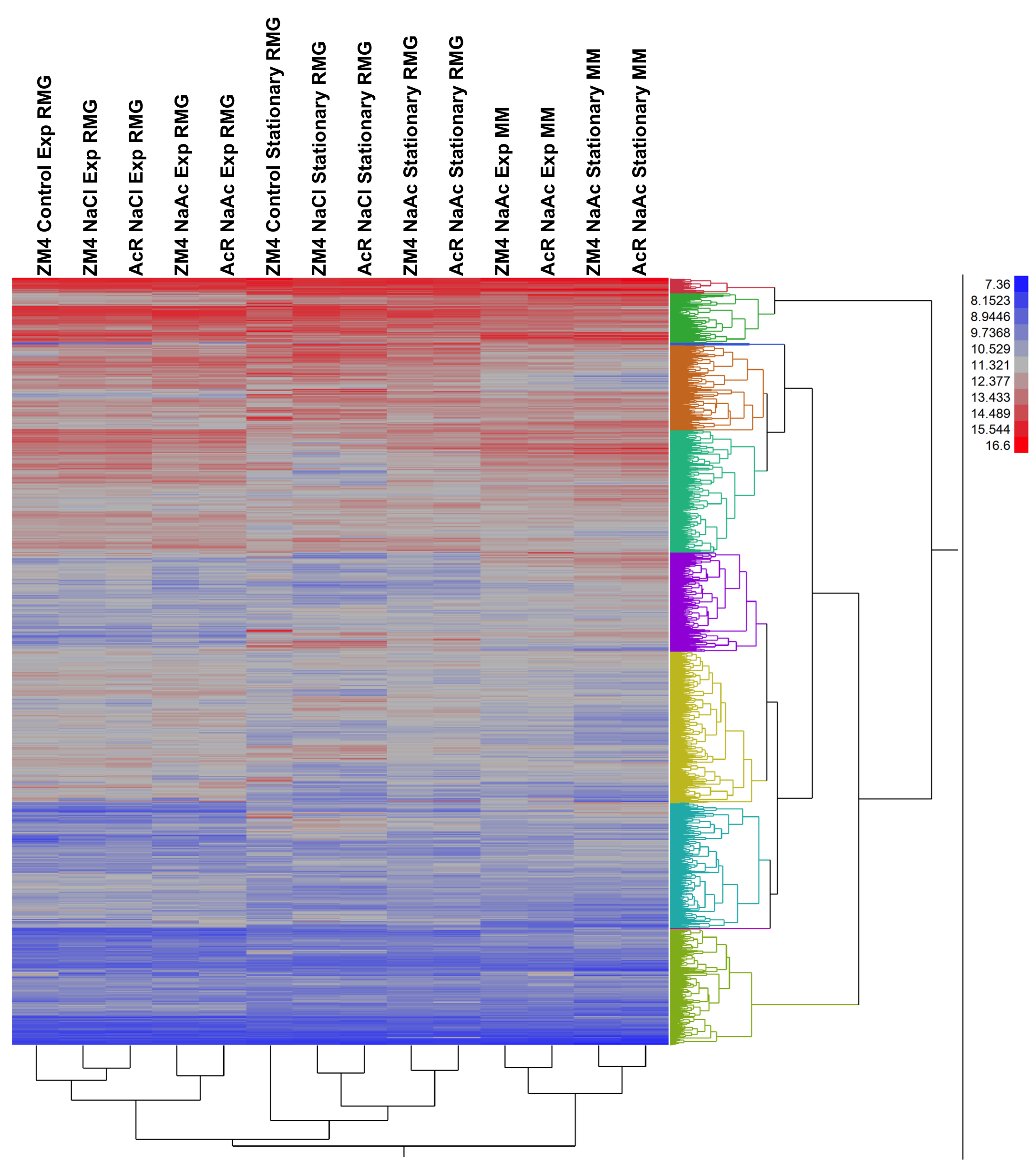

Supplement: Supplementary file 1 [file DataSheet1.DOCX]
